# Biofilm Analysis by Confocal Microscopy—Basics and Practical Aspects

**DOI:** 10.1002/jemt.70064

**Published:** 2025-09-10

**Authors:** Thomas R. Neu, Ute Kuhlicke

**Affiliations:** ^1^ Department of River Ecology Helmholtz Centre for Environmental Research–UFZ Magdeburg Germany

**Keywords:** applied aspects, bio‐aggregate, biofilm, CLSM, confocal, fluorescence staining, sample mounting

## Abstract

This review is intended as a guideline for beginners in confocal laser scanning microscopy. It combines basic theoretical concepts, such as fluorescence principles, resolution limits, and imaging parameters with practical guidance on sample preparation, staining strategies, and data acquisition using confocal microscopy. The aim is to combine technical and methodological aspects in order to provide a comprehensive and accessible introduction. The main application is in microbiology, with a focus on biofilms and bio‐aggregates; although other researchers in biology may benefit from this survey. In this primer, we compiled 25 years of experience with confocal microscopy, four generations of instruments, diverse national and international projects, and many different samples of PhD students, PostDocs, and senior scientists from various countries.

## Graphical abstract

Development and detachment of an interfacial microbial community. The resulting biofilms and bio‐aggregates are three‐dimensional communities susceptible to examination by confocal laser scanning microscopy.

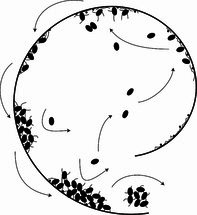

**Summary**

- The manuscript represents a general guideline for beginners of confocal microscopy, though mainly for microbiologists.
- As numerous microbial samples are three‐dimensional, the focus is on multichannel analysis of hydrated biofilms and bio‐aggregates.

## 1 LSM Introduction

The key for understanding and applying confocal laser scanning microscopy (CLSM) is based on the knowledge of fluorescence, specimen preparation, digital and confocal imaging. Consequently, the user should become familiar with light microscopy in general and the basics of fluorescence and fluorescence microscopy. In fact, epifluorescence microscopy represents the perfect technique if the sample is thin enough (few μm), for example, for suspended bacterial cells or bacterial cell monolayers. However, most biological objects are thicker and thus three‐dimensional, including microbial biofilms and bio‐aggregates, resulting in blurred images due to signal from out of focus planes. This problem tackled in the 70s and 80s resulted in the development of CLSM. By this means, the step from two‐dimensional imaging to three‐dimensional imaging became possible, including an improved resolution. Although transmission and scanning electron microscopy offer a higher resolution, the disadvantage comprises the necessity for fixation and dehydration of the biological sample. Thus, CLSM with one‐photon excitation (confocal) allows three‐dimensional examination of thick and fully hydrated samples with signals recorded in multiple channels. A laser microscope with additional multi‐photon excitation enables imaging in even deeper locations and offers some additional features. The resolution of CLSM determined by the law of Abbe is dependent on the wavelength of light, refractive index of the medium, and the semi‐aperture angle of the objective lens. Roughly, this means half the wavelength of light used for imaging (about 250 nm). However, in the meantime, new techniques and instrumentation allow doubling of this resolution. On top of that, recent developments revealed approaches and instruments with even higher resolution, overcoming the diffraction barrier. These fluorescence‐based techniques are summarized with the term nanoscopy or super‐resolution techniques resolving structures in the few nm range. In addition, new optical architectures allow three‐dimensional imaging of larger areas with reduced bleaching of samples.

Although this review has a focus on CLSM, the additional laser microscopy techniques are also briefly mentioned, which might be available in certain facilities (spinning disk laser microscope) or at combined instruments (two‐photon laser). In any case, the basic and applied aspects discussed will be applicable to these instruments as well. Finally, some emerging high‐resolution techniques are mentioned that any CLSM user should certainly be aware of.

## 2 What Can CLSM Offer for Biofilm Studies?

This paragraph should begin with a citation attributed to Aristotle (384–322 bc): “thinking is impossible without an image.” This brings the essence of imaging by CLSM to the point. The image will support ones thinking about a particular biofilm structure or a biofilm experimental design as well as how to measure certain parameters of a microbial biofilm. The images derived from confocal datasets can reveal the three‐dimensionality of the biofilm architecture, overall composition of the biofilm, bacterial cell distribution, identity of procaryotes, presence of eukaryotic microorganisms, biofilm matrix type and distribution, porosity of the matrix, several metabolic features as well as activity of specific genes. In summary, the multichannel image datasets allow to analyze a number of important parameters of a defined biofilm system regardless whether immobile (biofilm) or mobile (bio‐aggregate). Thus biofilm CLSM in short, the hydrated biofilm sample is optically sectioned in multiple channels at a defined step‐size resulting in a series of optical thin sections which can be projected and analyzed as a three‐dimensional data set.

## 3 The 10 Commandments

For beginners to get going, the so‐called 10 commandments stated in one of the basic confocal handbooks may be helpful as a start to find the right attitude:

Ten commandments according to Jerome and Price (Jerome and Price 2018)

1. The perfect microscope and perfect microscopist do not exist
2. Confocal microscopy is more than a confocal microscope
3. Specimen processing—integrity must be maintained
4. Photons are your friends and signal‐to‐noise ratio is king
5. Quantification is a challenge and at best semi‐quantitative
6. Scientific digital imaging and normal photography are not the same (data versus photos)
7. Images are your data: garbage in = garbage out
8. Resolution and bit depth in a digital image are set after recording
9. JPEG file format (compressed) is EVIL
10. Storage media is essentially free and infinite

## 4 Fluorescence and CLSM Basics/Theory

### 4.1 Visible Part of the Electromagnetic Spectrum

**FIGURE 1:**
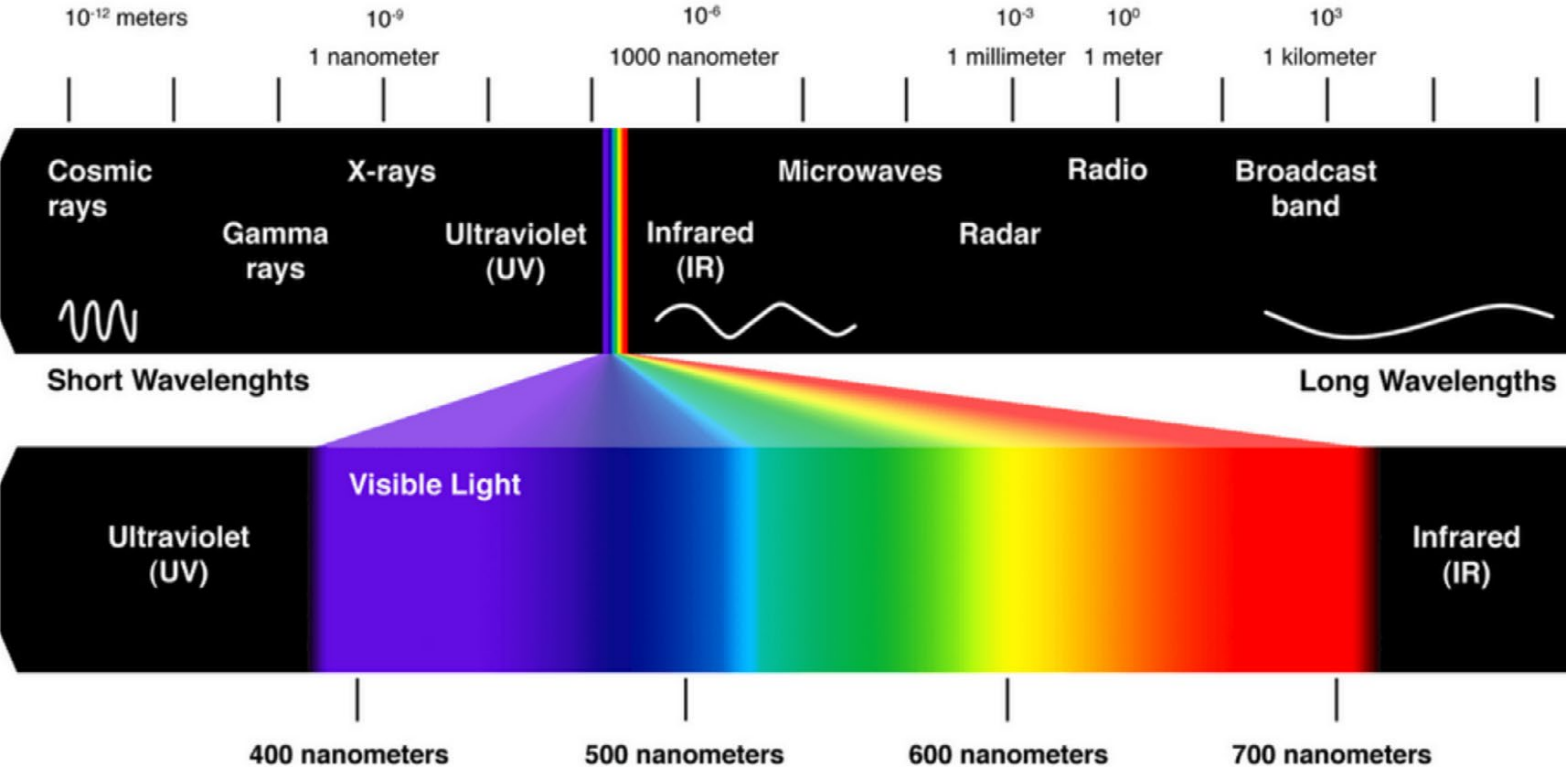
Electromagnetic spectrum showing the visible range. *Source:* Quora.

The visible part of the electromagnetic spectrum ranges from about 380 to 750 nm from violet, blue, green, yellow, orange, red to far red (Figure 1). This range covers the most frequent applications in epifluorescence and CLSM. Shorter wavelengths in the UV (360 nm) are necessary for optimal excitation of UV fluorochromes. Longer wavelengths in the near‐infrared (750–1400 nm) are important for near‐infrared fluorochromes and especially for multi‐photon excitation. Both UV and IR require expensive additional laser hardware and are thus not readily available. In addition, special filters and objective lenses are needed. Due to the properties of organic fluorochromes in terms of their absorption and emission spectrum, only a limited number of stainings are possible within the visible range. In fact, three or at maximum four fluorochromes fit into this range based on the width of their emission signals. Even so, the tail of each emission spectrum may result in an overlap with subsequent channels, a phenomenon called bleed through or crosstalk (see later).

### 4.2 Fluorescence Excitation and Emission

**FIGURE 2:**
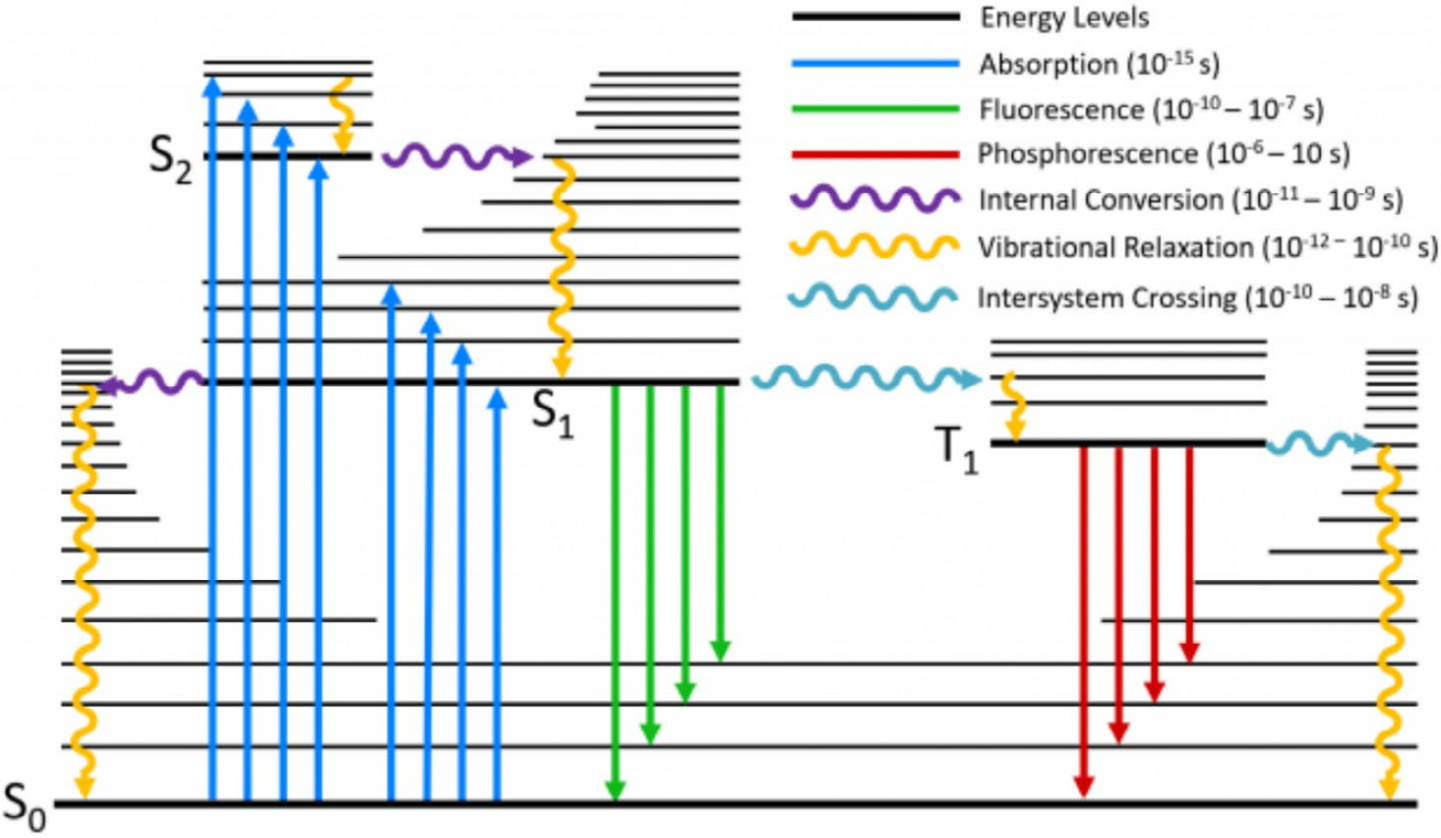
Jablonski diagram showing absorption and fast emission (fluorescence) as well as time delayed emission (phosphorescence). *Source:* Edinst.

**FIGURE 3:**
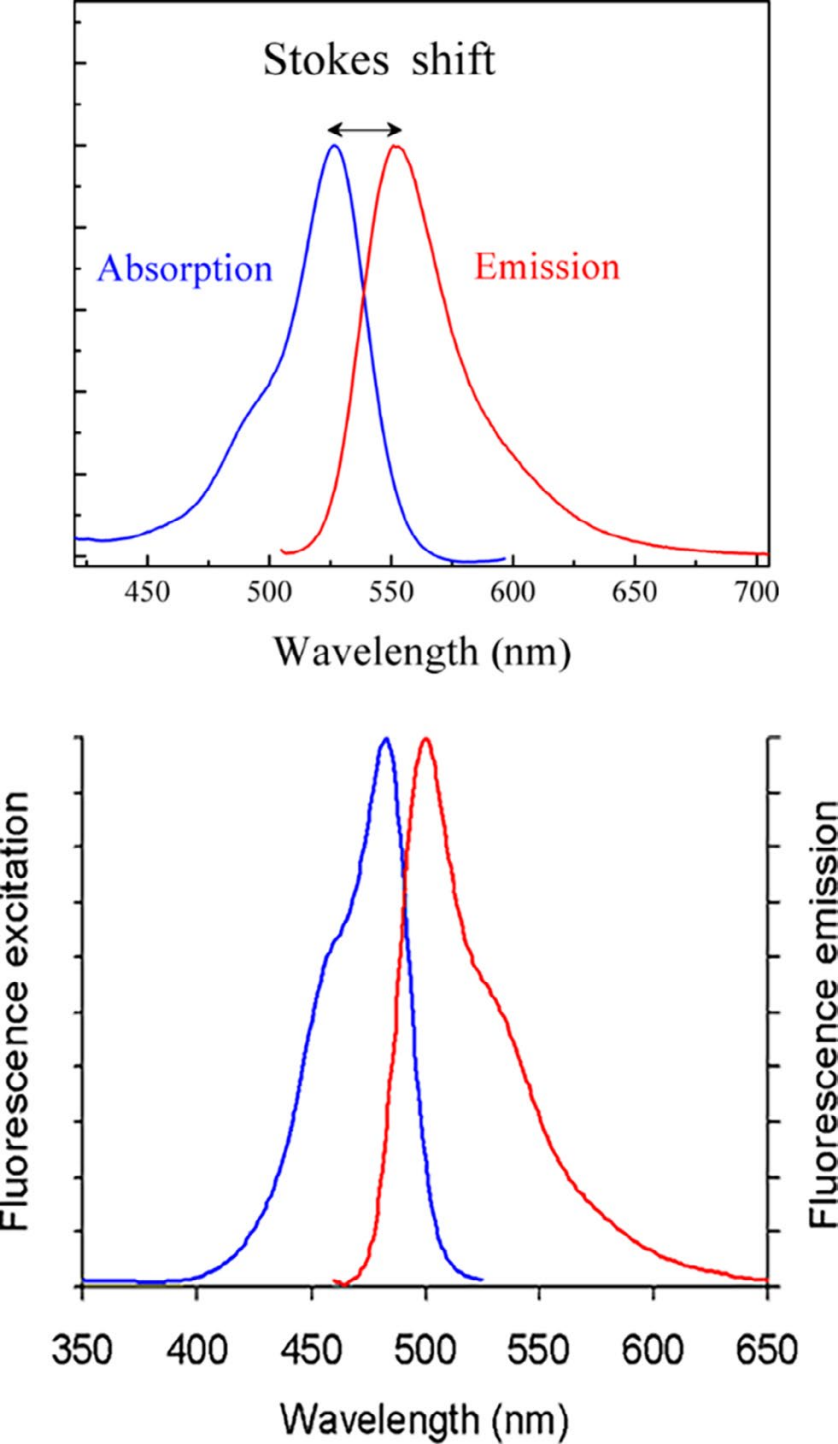
Absorption and emission spectrum of a hypothetical fluorochrome indicating the Stoke shift (Top). Usually with most fluorochromes, the Stoke shift is much smaller. *Source:* Wiki. Absorption and emission spectrum of a real fluorochrome, Syto9 (Bottom). Please note the very narrow Stoke shift and the tail of the emission spectrum towards the longer wavelengths.

A fluorochrome as an organic molecule with double bonds able to absorb a single photon at a certain energy λ_1_ (shorter wavelength) becomes excited and after a short time emits most of this energy at a different energy level λ_2_ (longer wavelength) meaning λ_2_ > λ_1_. The difference in energy is due to internal non‐radiative relaxation processes. This process can be shown using the so‐called Jablonski diagram (Figure 2). For example, blue excitation (488 nm) of Fluorescein isothiocyanate (FITC) or Syto9 (nucleic acid stain) results in a green emission signal in the range of about500 to 550 nm. The difference in wavelength between maximum excitation and maximum emission, called Stokes shift, represents a key characteristic of each fluorochrome. However, for practical use, not only are these maxima of interest, it is also crucial to know the complete absorption and emission spectrum of a fluorochrome (Figure 3). This is important for knowing the degree of excitation at a certain wavelength, which determines the emission signal intensity and for defining the detection range of the emission signal as well as to identify possible crosstalk. For this purpose, a number of spectra viewers are available online (see table at the end with Supporting Information).

### 4.3 Technical Terms to Understand

**FIGURE 4:**
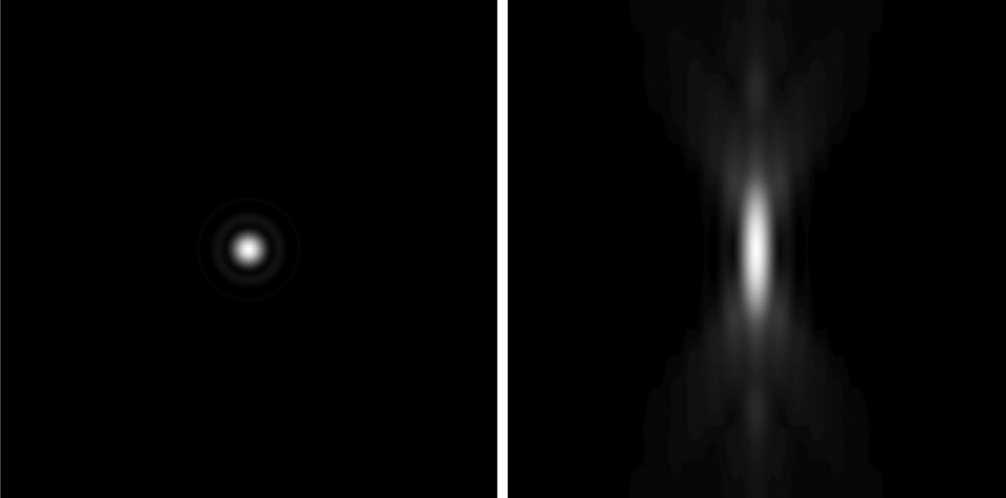
Point spread function of a fluorescent bead in lateral *XY* direction (left) and axial *Z* direction (right). *Source:* Zeiss.

The point spread function (PSF) describes the light intensity distribution of an object in an image or, in other words, describes the imaging properties of an instrument and objective lens. The PSF in the *XY* direction is acceptable, but the PSF in the *Z* (axial) direction shows an elongation in dependency on the numerical aperture (NA) of the objective lens (Figure 4). The higher the NA, the shorter the axial elongation and the higher the resolution in both the *XY* and *XZ* directions.

**FIGURE 5:**
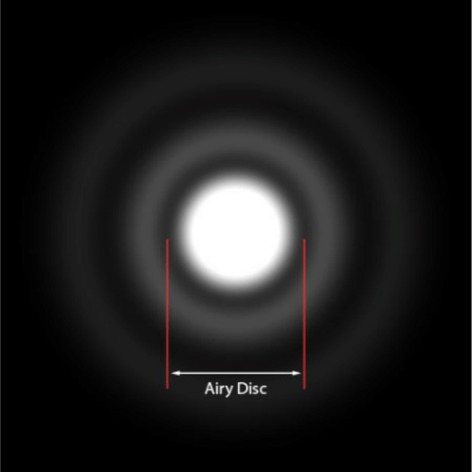
Point spread function in the focal plane showing the inner ring defined as Airy = 1. *Source:* Introduction to Optics. 3rd ed., Pedrotti et al. Cambridge University Press, 2017.

The Airy disc (after George Airy) represents the PSF in the focal plane (Figure 5). The inner ring of the Airy disc is equal to a pinhole setting of Airy = 1, which is a setting in the microscope software for standard imaging. If two objects (Airy disks) are moved closer together, at a certain distance, the two objects cannot be resolved. The resolution is lost when the inner ring of Airy disk 1 is at the maximum of Airy disk 2.

As indicated, the law of Abbe describes the lateral resolution δ of the CLSM according to the following equation:

$$\delta =\frac{\lambda }{2n\times \sin\alpha }$$

with λ as wavelength, *n* as refractive index of the medium, and α as semi‐aperture angle of the objective lens.

The numerical aperture (NA) of an objective lens represents a  ajor parameter defining the resolution of the lens. The NA of an air lens is usually low, whereas immersion lenses have a higher NA in the order of water, glycerol, and oil. A high NA means a value of 1.2 (water immersion), 1.3 (glycerol) or 1.4 (oil immersion). The definition of the NA is as follows:

NA=n×sinα

The Nyquist criterion frequently comes up if one is working at the CLSM. The Nyquist criterion determines the minimal sampling density required to capture and transfer all information from the microscope (object) into the image. If the sampling density is larger than Nyquist, some information of the sample is lost (undersampling), if the sampling density is higher than Nyquist to much information (data) is recorded (oversampling). According to Nyquist, in a typical CLSM setting the lateral resolution should be 50 nm (pixel size) whereas the axial resolution should be about 160 nm (step size). For practical imaging, this means a high degree of bleaching, thus many users do not record their image data sets along the Nyquist criterion. However, circumventing the problem is possible by: 1) using an extremely stable fluorescent probes such as quantum dots (see later), or 2) recording data at a lower signal to noise ratio in combination with subsequent image restoration (deconvolution) which is explained further down. Information on the topic “Nyquist” and “sampling” found at the “Scientific Volume Imaging” (SVI) website will explain many more details.

### 4.4 Widefield Epifluorescence Microscopy Versus Confocal Microscopy

**FIGURE 6:**
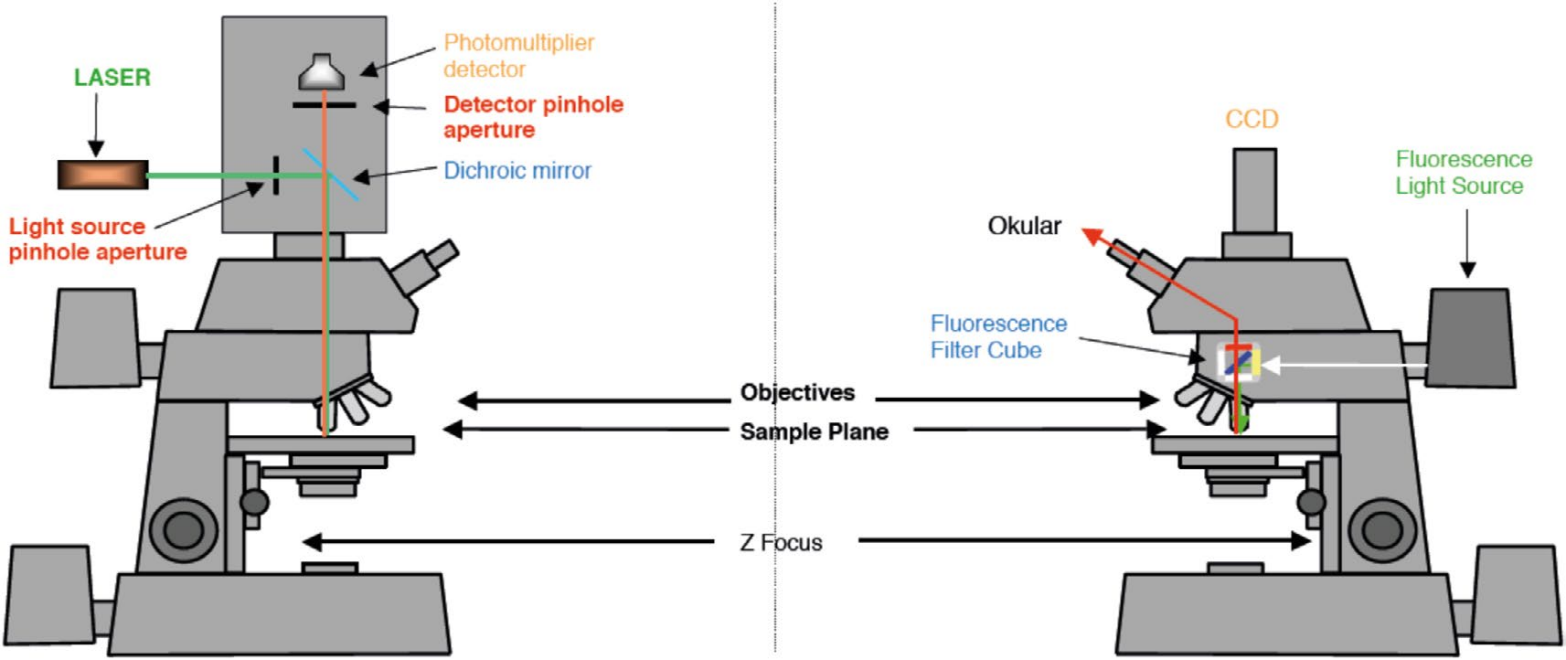
Comparison of confocal laser scanning microscopy (left) with epifluorescence microscopy (right). *Source:* www.zmb.uniz.ch .

The widefield epifluorescence microscope, upright or inverted, is an integral part of every confocal microscope setup. In fact, the usual procedure is that a sample is examined first by visual observation in the epifluorescence mode. That means excitation will be done by using the illumination of the epifluorescence microscope (e.g., metal halide lamp) in combination with the appropriate filter selecting a specific color. For splitting the excitation light from the emitted light, again the filter cubes of the epifluorescence microscope are employed. At that stage, no laser is involved. After visually selecting a spot for imaging, the instrument is switched to the laser mode. Only then will the excitation of the fluorochromes be by the matching laser line selected, and at the same time, the beam splitter and filters of the scan head are engaged for imaging. The scanned image recorded by the detector, often a photomultiplier, will be transferred onto the monitor of the CLSM instrument. In short, the CLSM can be used in the epifluorescence mode for visualization by eye and in the laser scanning mode for recording data sets via the detector (Figure 6).

### 4.5 Confocal Principle

**FIGURE 7:**
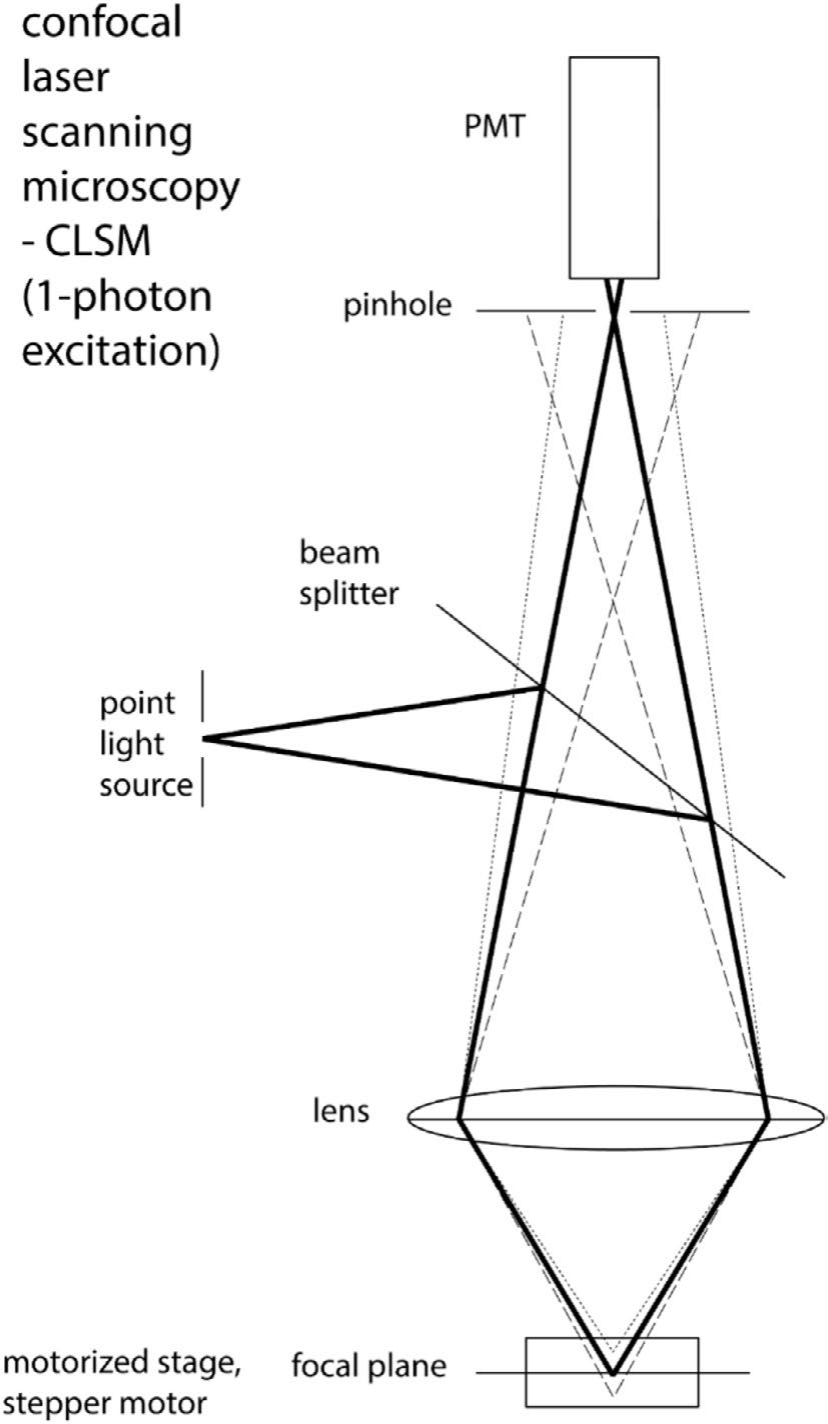
The key elements of a CLSM with point light source, pinhole in front of the detector, and motorized stage with stepper motor.

The principle of CLSM based on three key components comprises: (1) a point light source (laser) with an illumination aperture for excitation, (2) a confocal aperture (pinhole) in front of the detector, and (3) a stage with a stepper motor for moving the sample in the axial direction (Figure 7). By establishing this optical geometry, the CLSM has the following characteristics: (1) point‐by‐point illumination of the sample, (2) nearly complete removal of out of focus light, and (3) optical sectioning of the object. The term “confocal” in the acronym CLSM originates from the fact that the pinhole is conjugated to the focal plane of the objective lens. This optical geometry results in a number of advantages but also some limitations compiled in Table 1 including comments.

**TABLE 1:** Major advantages and disadvantages of CLSM.

Advantages	Comments
Investigation of fully hydrated living microbial communities	In contrast to electron microscopy were fixation and dehydration is necessary
Examination of samples with more than 100 μm thickness	Depending on scattering and absorption of the sample, the imaging depth can be from 10 μm up to 2 mm
Reduction of scattering by point illumination	Due to optical geometry
Capability of non‐invasive sectioning by virtually no out of focus blur	The dimension of out of focus blur is depending on the pinhole setting, with Airy = 1 as the best compromise
Possibility of horizontal (XY), vertical (XZ) and temporal sectioning	XY scanning is mostly used, XZ means a side view of the sample which however will show signal elongation
Simultaneous application of multiple probes and multi‐channel recording of digital enhanced signals	If multiple probes are used often a sequential scan is compulsory
3D analysis and organization of macromolecules, single cells, micro‐colonies and complex microbial communities	Best results are achieved with advanced digital image analysis software
**Limitations**	**Comments**
One‐photon bleaching and cell damage in out of focus areas	Although the sample is imaged in the focal plane only, some light will reach the area above and below
Depth of laser penetration	The shorter the wave length the more reflection occurs, biological samples absorb light
Optimal adjustment of signals if large differences in emission intensities	Using specific lookup tables the optimal signal to noise ration cannot be adjusted across the field of view, it can be optimized only for the bright objects or for the faint objects
Toxicity of fluorochromes	The effect of fluorochromes on living organisms is largely undetermined
Fluorescence of background	Might be the case with biofilms on mineral surfaces and leaves or if non‐transparent plastic is used as substratum for biofilm growth
Mobility of microorganisms (if point scanner)	Motile cells will create a streak in the image when the scan runs, in this case a spinning disk microscopy should be used

## 5 CLSM Instrument

### 5.1 CLSM Variations and Architecture

**FIGURE 8:**
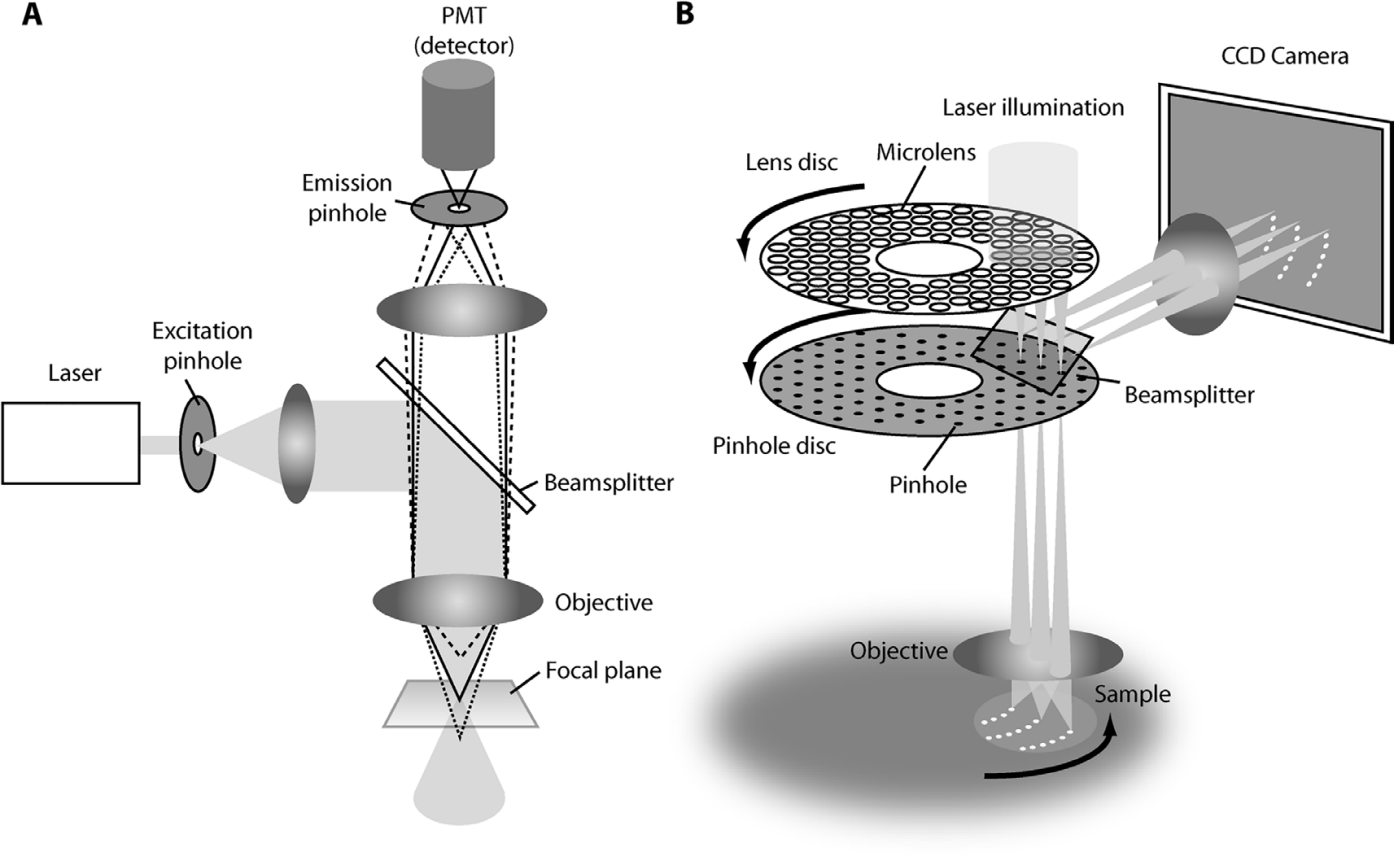
Comparison of confocal laser scanning microscopy with spinning disk laser scanning microscopy. From Gräf et al. (2005).

There are two types of confocal instruments, point and disc scanning laser microscopes (Figure 8). Disc scanning microscopes scan at high speed and consequently are suitable for samples with motile objects. The current spinning disc microscopes are built with two discs, one with an arrangement of micro‐lenses and another one with corresponding pinholes. The architecture is patented as Yokogawa spinning disc scanner and available from different suppliers. The point scanning microscopes are more widely distributed as they achieve a higher resolution. According to its designation, the scanner moves the laser beam point by point and line by line in the *XY* direction. Apart from the recording speed, the major difference is the fixed pinholes in spinning disc microscopes in contrast to a (mostly) single variable pinhole in point scanning microscopes. In any case, recent developments show that different architectures and improvements in hard‐ and software as well as combinations of techniques allow even more improved resolution when compared with traditional early CLSM's before the turn of the century.

The standard CLSM instrument represents a compilation of several devices mounted around an optical table. The essential basis represents an epifluorescence microscope, which can be upright or inverted. The upright stand allows mounting of many different sample types, whereas the inverted stand is ideal for culture dishes with an optical window (coverslip quality) at the bottom. The microscope stage usually consists of two motorized tables, a large one used for coarse movements (μm) as well as a fine‐tuned one for steps in the tens of nm range. In the epifluorescence mode, the sample is examined visually using the standard light source (mercury lamp, metal halide lamp or light diodes) and filters of the epifluorescence microscope for excitation and observation. With state of the art instruments, these filters are moved by a motor using a touch pad at the microscope. The lasers are attached to the epifluorescence microscope via the scan head. The scan head also contains the filters for excitation, the scanner, a beam splitter, the filters for emission, and a variable number of detectors. Some instruments have an option of scanning at normal speed as well as a so‐called resonant scanner for fast scanning and imaging. The various CLSMs are also different in their beam splitters. There are traditional beam splitters but also acousto‐optical beam splitters which result in higher transmission (stronger signal) and allow simultaneous separation of several excitation and emission lines.

### 5.2 Lasers

The continuous wave lasers attached to a CLSM can be gas, diode, or fiber lasers with a single or a few laser lines. Employing a combination of standard lasers (e.g., Argon, DPSS, and HeNe) with lines at 488, 561, and 633 nm will allow for three‐channel imaging of the traditional fluorochromes FITC, TRITC, and Cy5 or other fluorochromes with similar excitation/emission spectra. Nevertheless, there are other lasers with different excitation lines that can be combined depending on the main application. State of the art are nowadays pulsed lasers such as white lasers also called supercontinuum light sources. They offer the full spectrum of visible laser light from 440 to 790 nm for excitation. In this range, up to eight laser lines can be used simultaneously. Other pulsed lasers in the near IR for multiphoton laser microscopy represent a special add‐on suitable for imaging at deep locations and other specific applications (see section below). The laser light is guided into the scan head via mirrors, a tube containing a special liquid, or a glass fiber cable. The filters in the scan head are similar but different compared to the filters used in the epifluorescence mode for visual examination of the sample. As a result, the visible examination of the sample does not fully match the laser‐based image on the computer monitor.

### 5.3 Objective Lenses

For the manyfold applications, there are numerous objective lenses available. They differ in terms of magnification, numerical aperture, correction, immersion media, coverglass correction, and free working distance. Of note, magnification is not that important, as it only defines the area of the sample examined. Most important is the numerical aperture (NA) as this parameter only determines the resolution. The lenses with the highest numerical aperture (NA 1.4) require oil immersion, but they have a short working distance (about 100 μm). For many applications, water immersion lenses with a high numerical aperture (NA 1.2) are extremely useful, as they show a bright signal and have a longer working distance (220 μm). As a consequence, they are easier to work with and are less messy. There are lenses to be used with or without coverglass. They are called water immersion lenses if a coverslip is required and water immersible or dipping lenses if no coverslip is used. The latter ones are ideal for imaging larger samples having a strong topology, such as environmental biofilms. Further, these lenses have an extra‐long working distance in the mm range, making them very convenient to work with. Some lenses optimized for imaging at short (UV) or long (IR) wavelengths are available if applying the appropriate fluorochromes. Details of the objective lenses and their applicability found at the websites of microscopy companies show further properties, their detailed characteristics, and usual applications.

### 5.4 Detectors

Standard detectors are multiple photomultiplier tubes (PMT) with a rather low quantum efficiency or one charge‐coupled device (CCD) having a much higher quantum efficiency. Several PMTs are built into conventional CLSM as they are cheap, whereas a single CCD camera is usually attached to a spinning disc laser microscope. Nowadays, often hybrid detectors, showing a higher quantum efficiency than traditional PMT's, are built into the scan head. It is important to know that the detectors record pixel intensities, for example, for 8‐bit data in 256 different gray values from 0 = black (background) to 255 = white (saturated) or for 12‐bit data gray values from 0 = black to 4096 = white (scientific standard). Thus, all the colorful LSM images presented show allocated false colors. Sometimes the colors match the emission signal seen in the microscope, but they may also be a completely different color as the monitor works in the red‐green‐blue (RGB) mode and the resulting overlay colors.

### 5.5 CLSM Instrument Control

Attached to each CLSM instrument is a high‐end PC together with one large or two small monitors in combination with the proprietary software for controlling the microscope. Handling the CLSM uses the mouse and keyboard and sometimes an additional control panel having manual knobs for different major functions. The software of any CLSM is highly complex and allows one to run the instrument in a smart and quick mode but also in an advanced mode for experienced operators adjusting each of the individual parameters separately (see below). Many laboratories use the CLSM setup only for recording image data sets, whereas the analysis and visualization of image data is detached and done at a different computer with other software packages specific for visualization, deconvolution, or semi‐quantification.

## 6 Samples

### 6.1 Mounting

**FIGURE 9:**
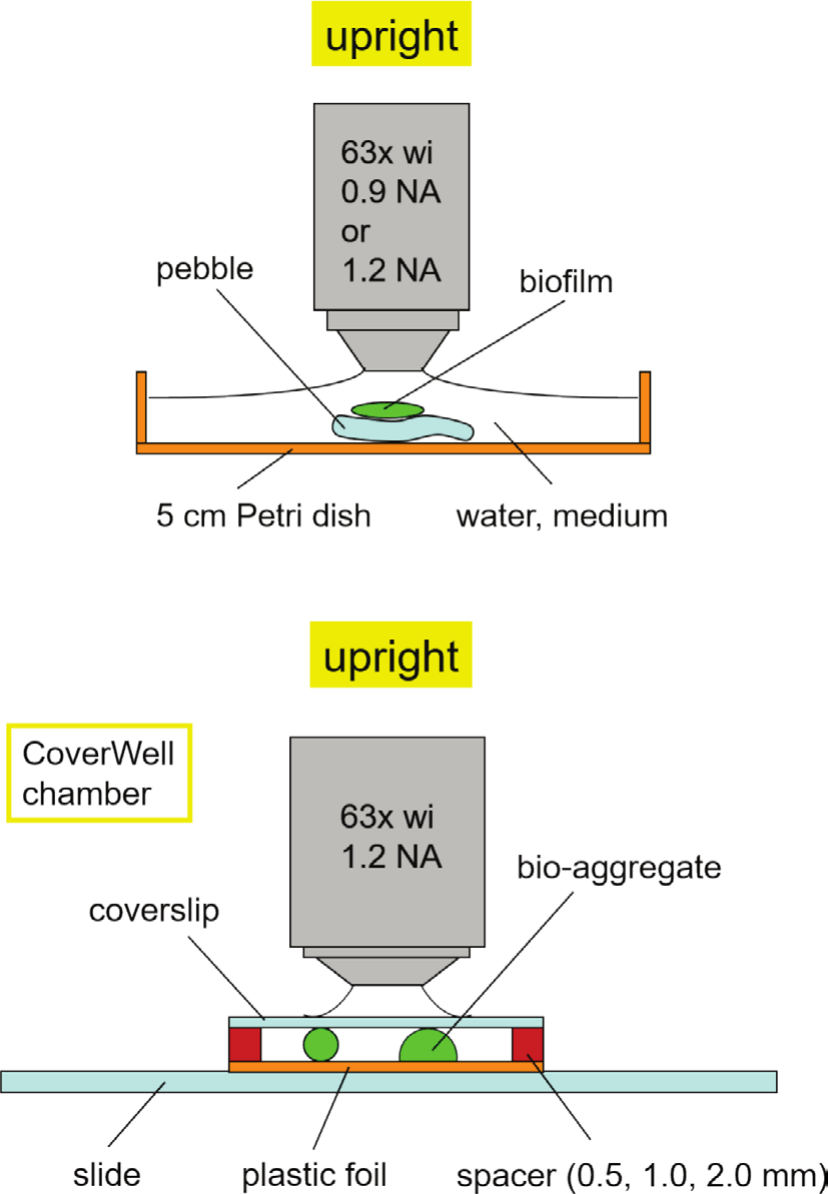
Two popular mounting options for the upright microscope. The Petri dish method is ideal for larger samples and biofilms on a surface (top). The CoverWell chamber approach is more suitable for more fragile bio‐aggregates and granules (bottom).

**FIGURE 10:**
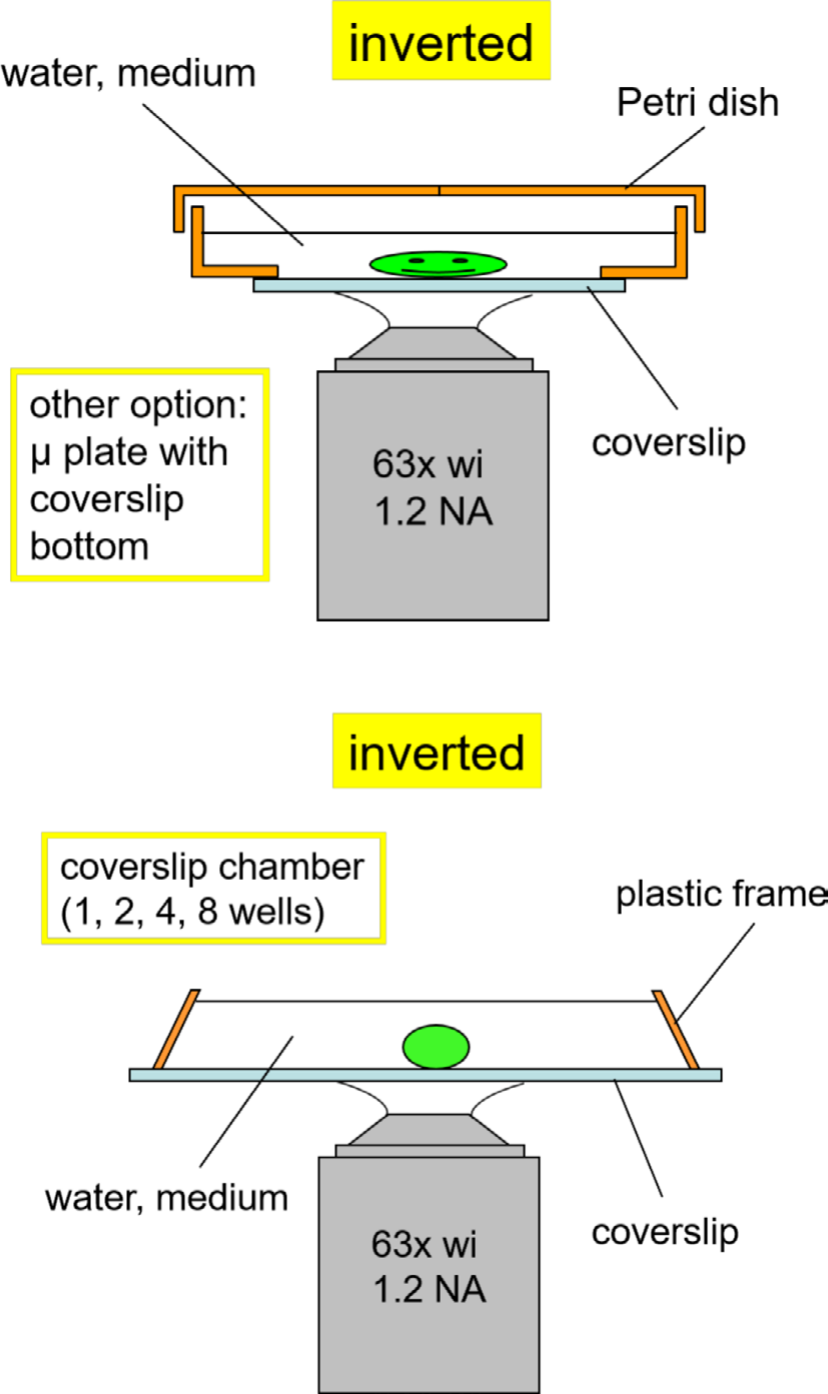
Two mounting options for the inverted microscope. The Petri dish method is ideal for larger samples (top). The coverslip chamber approach is more suitable for more fragile bio‐aggregates and granules (bottom).

Just about any biofilm sample fits under the CLSM if the upright microscope stand is available. In most cases, the limitation is the weight of the sample and the space between the microscope stage and objective lens. The weight is critical for precise positioning of the sample on the microscope table during the scan. In fact, this can be a real problem if the motor cannot keep the adjusted position and focus during the scan in three dimensions. More recently, there are new types of laser microscopes and technical measures that circumvent this restriction. Biofilm samples might be on natural surfaces (e.g., minerals, wood, or leaves) or manmade surfaces (e.g., glass, plastic, concrete, or other defined material). The main strategy in CLSM is aimed at mounting without disturbing the biofilm or bio‐aggregate structure. In contrast to electron microscopy, this mounting is done in the fully hydrated state. For the upright microscope, the sample is mounted in an open Petri dish or in a chamber with a spacer (Figure 9). After mounting and staining in a Petri dish, examination using long working distance water immersible lenses is most convenient. For mobile biofilm systems, such as bio‐aggregates, flocs, or granules, mounting in CoverWell chambers with the matching spacer in order to avoid squeezing the fragile structure represents the method of choice. These chambers, closed with a high‐quality coverslip (No. 1.5H) require examination with water immersion lenses. If an inverted microscope is available, the chambers ideally have a high‐quality coverslip bottom. The devices comprise special Petri dishes, coverslip chambers, or so‐called μ plates (Figure 10).

### 6.2 Sample Properties

The general strategy for imaging is always: first take advantage of sample properties without staining. Very often biofilm samples show a reflection signal from the surface to which they are attached. In addition, embedded particles from the environment but also microbial cells show a certain degree of reflection. For example, some bacteria have inclusion bodies showing a strong reflection (e.g., sulfur granules). This signal is for free and very often neglected. Reflection of the laser beam is recorded at the point of excitation usually plus minus 5 nm. Of notice, this option is not available at each instrument.

In environmental biofilms, the presence of light results in a mixed phototrophic microbial community. The associated photo pigments, such as chlorophyll A (algae and cyanobacteria) or phycobilins (cyanobacteria) show a very strong autofluorescence signal. These two pigments help to locate and differentiate algae and cyanobacteria based on their autofluorescence in two different channels in the orange and far‐red part of the spectrum. Cyanobacteria sometimes show an additional green autofluorescence originating from a UV protecting pigment (scytonemin). Depending on the type of microorganisms, there might be additional autofluorescent constituents useful for imaging. For example, methanogenic bacteria have the cofactor F_430_ which can be excited in the UV and used for imaging.

For certain samples, the CLSM with an appropriate detector can be run in the transmission mode, which is useful, for example, for imaging thin samples containing protists or fungi. Of note, the transmission signal is not confocal. As a take‐home message, reflection and transmission imaging as well as possible autofluorescence should be explored before considering staining the sample. In fact, the autofluorescence of a sample has to be known as a control in order to select fluorochromes with an emission in a different part of the spectrum.

Sometimes the samples are very thick (mm‐cm) such as microbial mats, or if biofouling samples have to be examined. Consequently, the sample has to be embedded and physically sectioned. A key issue with all embedding techniques is sample integrity. For biofilms and bio‐aggregates, several embedding techniques have been described using agarose, acrylamide, or nanoplast (Christensen et al. 1999; Decho and Kawaguchi 1999; Droppo et al. 1996). Staining of the sample can be done before or after embedding, depending on the permeability of the probe into the embedding material. For sectioning of samples, a scalpel or razor blade can be used. If available, a microtome will produce (after some training) perfect sectioning results. Cryo‐sectioning represents another option for which the sample is embedded into a special cryo‐gel. However, be aware that the cryo‐sections will thaw when they are collected on a slide after sectioning. This may cause some artifacts and may disturb the original structure.

## 7 Contrasting Agents

### 7.1 Fluorochromes and Fluorescent Probes

For contrast, low molecular weight organic fluorochromes, high molecular weight probes (e.g., proteins) conjugated with fluorochromes, and nanoparticles are available. In many cases, staining with low molecular weight fluorochromes specific for biochemical compounds such as nucleic acids, proteins, or membranes allows targeting bacterial cells throughout a biofilm sample. For each of them, there are several options having different excitation and emission ranges, which have to match sample properties. For example, if a biofilm contains microalgae, they contain chlorophyll A, showing an emission signal in the far red part of the spectrum. Consequently, a fluorochrome emitting in the far red cannot be applied.

Classical nucleic acid‐specific fluorochromes comprise acridine orange (AO) and 4′,6‐Diamidin‐2‐phenylindol (DAPI). However, for confocal work they are critical as both have a very broad emission spectrum, and DAPI ideally requires a UV laser for excitation. Furthermore, they may also stain other constituents which are not nucleic acids.

In the meantime, newly developed nucleic acid‐specific fluorochromes such as the Syto series are quite popular. Syto's are available in different colors for live cell staining. More sensitive is SybrGreen, which initially was used for viruses, but often shows rapid bleaching. For fixed samples, the nucleic acid‐specific Sytox series was developed. Other biochemical targets are proteins stained with one of the Sypro fluorochromes or lipids stained with FM fluorochromes.

There are also a number of fluorescent stains for eukaryotic microorganisms available. Their cellular organelles are mostly the target for staining. The fluorochromes can be used for staining the nucleus, mitochondria, lysosomes, endoplasmic reticulum, and Golgi apparatus. However, be aware that some of these fluorochromes will also stain bacteria.

Fluorochromes attached to other proteinaceous probes offer a wide area of applications. For example, antibodies against all kinds of structures labeled with fluorochromes can be used in various ways. Similarly, lectins as glycoconjugate‐specific proteins labeled with a fluorochrome are very useful. For all of these proteins, there are labeling kits with different colors available from several companies.

Other popular probes cover the wide range of fluorescent proteins as a label, with the green fluorescent protein (GFP) most frequently used. Inserting the gene for GFP with molecular techniques into the microbe of interest and subsequent expression of the GFP product allows detection by CLSM without staining. In the meantime, there are many different fluorescent proteins available, such as YFP, RFP, or mCherry, and so on. Sure enough, inserting multiple genes for different fluorescent proteins in one microbe at different locations in the genome allows the study of their location in the biofilm as well as a specific gene function or activity simultaneously and over time.

A further potential fluorescent label are so‐called quantum dots (Nobel Prize in Chemistry 2023). The Q‐dots are semiconductor nanocrystals with several advantages, including high quantum yield (bright emission signal) and extreme photo‐stability (no bleaching). In addition, they are available in different colors. If necessary, several different types of Q‐dots can be excited using a single wavelength, and they have a very narrow emission signal. Thus, in contrast to organic fluorochromes, more probes can be applied in combination within the visible part of the spectrum. The drawbacks are their toxicity and that they are not water‐soluble, and as a consequence, they need an organic coating. Despite this, they are offered for linking to proteinaceous probes as a very bright and stable fluorescent label.

Finally, it is worth mentioning that the appearance of a microbial cell is dependent on the site of staining. For example, a DAPI‐stained cell will look very tiny due to the interior staining target in comparison to a cell stained at the cell surface, for example, with Sypro. In any case, apart from the type of fluorochrome or probe, consideration of the probe size with respect to the target to which they bind becomes also important.

### 7.2 Staining Strategy

In order to approach a new sample, there is a logical follow‐up strategy for staining and examination by CLSM. It starts with sample properties, identification of biological components, activity measurements, probing the microenvironment, and in some cases taking advantage of GFP labeling. The strategy is compiled in the following Table 2.

**TABLE 2:** Staining strategy for a new sample and resulting information.

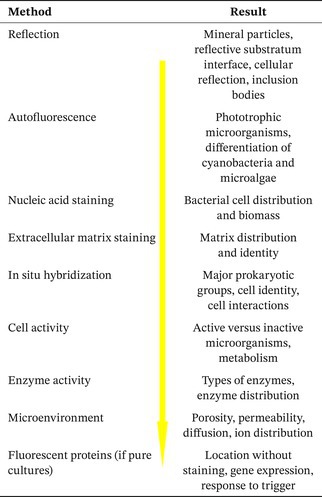

### 7.3 Staining Option and Protocols

As indicated, the sample has to be first examined without any staining. This is compulsory as a control, and the potential autofluorescence will determine the fluorochromes which can be applied. The reflection signal, although not possible in all instruments is “for free” and helps to put the microbial sample into a micro‐environment. Apart from fluorescence in situ hybridisation (FISH) all of the following protocols are fast staining techniques which require only 10–30 min.

Reflection—the signal is detected at the point of excitation. The reflection is strongest at shorter wavelengths. If a 488 nm laser is used, the setting for detection is defined at the same wavelength plus/minus 5 nm meaning from 483 to 493 nm.

Autofluorescence—this can be determined by examination of the emission signals in every possible channel, for example, in the green, orange, red, and far red. Ideally, the laser microscope allows a so‐called lambda scan where a single laser line (e.g., 488, 561, and 633 nm) is used, and the emission is recorded in small bands from the point of excitation to the end of the spectrum in the far red range. As a result, the optical window(s) showing no signal can be identified and used for staining. If a white laser is available, the sample can be examined with every possible laser line in order to identify the strongest excitation/emission.

Nucleic acid staining—the first stain applied to a microbial sample is usually a nucleic acid‐specific fluorochrome. In many cases, this will be a stain with emission in the green, such as Syto 9 or SybrGreen. The Syto series is available in several different variations/colors, which is useful for combined stainings, for example, with lectins (see below). The fluorochrome from the supplier should be divided into aliquots of 2 μL and stored in the freezer at −20°C. For staining, this aliquot is diluted 1:1000 with sterile water or buffer. For staining, the sample is covered with a few droplets of the solution and kept for 10 min in the dark at room temperature. The nucleic acid staining is fast and straightforward, as the background of the sample will be black and only the bound fluorochrome will show a fluorescence emission. Consequently, de‐staining or washing of the sample is not necessary. For paraformaldehyde (PFA) fixed samples, there are also nucleic acid‐specific stains available. A series called Sytox is offered with green or red emission.

Life/dead baclight staining—this rather popular staining combination has to be used with caution! For more references see the table with critical assessments of the technique (Neu and Lawrence 2016b). Basically, the staining with the Life/Dead kit is straightforward; it contains Syto9 as a live stain and propidium iodide (PI) as a dead stain. In fact, it means not necessarily dead cells but indicates cells with a compromised membrane. Thus, the live bacteria show a green emission and the compromised ones a red emission. However, there are a number of conditions that have to be carefully followed. Nevertheless, with appropriate controls, it may be used for pure culture studies. If the kit is applied to environmental cultures, the result may be rather inconclusive as the many different bacteria having different cell walls may stain in various ways. In the meantime, there are also other fluorochrome combinations reported with similar results and claims.

Protein staining—the Sypro series of fluorochromes is quite useful for staining cell surfaces. However, it was also proofed for imaging proteinaceous fine structures. Prepare aliquots of 5 μL and store at −20°C. Dilute the stock solution with 5 mL of filtered water or buffer. Most often SyproOrange or SyproRed are applied. After staining the samples should be incubated for 15 min in the dark at room temperature. Of note: so‐called “SyproRuby Biofilm Matrix stain” is promoted as stain for the extracellular matrix in biofilms. However, this is questionable as it represents a general protein stain similar to the other Sypro's and the biofilm matrix maybe composed of other polymers (polysaccharides, nucleic acids) as well.

Membrane staining—there are many lipophilic fluorochromes available. Two of them, so‐called FM stains, are especially developed for staining the membrane of cells. The FM 1–43 has a green emission, whereas FM 4–64 has a red emission. The stains were also applied for staining extracellular vesicles. Again, the stain should be stored at −20°C in aliquots of 10 μL. The aliquot is diluted 1:100 with water or buffer to get a suitable working solution. For staining, a few droplets are added to the sample and incubated for 10 min in the dark at room temperature.

Lectin staining—lectins are non‐immunogenic and non‐enzymatic proteins specific for glycoconjugates. Fluor labeled lectins represent a compromise to stain a part of the extracellular matrix of microbial communities. The reason for this is the fact that there is no general fluorescent stain for all polysaccharides. In fact, the lectins stain only a part of the polysaccharides or, more precise, a part of the glycoconjugates in the matrix. Many lectins are offered with a green fluorescent label, but other labels are also available. In addition, there are fluorescent staining kits which allow attaching any other color. Again, the lectins from the supplier are divided into aliquots of 100 μL and stored at −20°C. For staining, the aliquot is diluted 1:10 with sterile water or buffer. The sample is covered with a few droplets of the solution and kept at room temperature for 20 min in the dark. In this case, the sample has to be washed 3–4 times in order to remove the unbound lectins. Depending on the properties of the sample, this can be done in various ways (Neu and Lawrence 2014a). Usually, the lectin stained sample is counterstained with a nucleic acid‐specific fluorochrome having a different excitation and emission. The lectin approach was critically assessed (Neu et al. 2001) and applied to pure cultures (Neu and Kuhlicke 2017) as well as environmental biofilms (Neu and Kuhlicke 2022).

Multi‐channel imaging—as indicated, the lectin stain can be combined with a nucleic acid stain. Thus, the instrument has to be set for excitation with two laser lines and two emission ranges for recording the signals. For example: with Syto 9 excitation at 488 nm (blue), emission 500–550 nm (green), a lectin‐Alexa568 excitation at 561 nm (yellow), emission 580–620 nm (red). Similarly, the invers staining combination can be applied: a lectin‐Alexa488 excitation at 488 nm (blue), emission 500–550 nm (green), Syto 60 excitation at 633 nm (red), emission 650–720 nm (far red).

If the sample has several properties that can be employed for imaging, such as a biofilm or bio‐aggregate grown in a creek exposed to sunlight, the presence of phototrophs can be detected via their photo pigments. Then the following settings may be used: (1) reflection, excitation at 488 nm, emission 483–493 nm, (2) Syto 9 excitation at 488 nm (blue), emission 500–550 nm (green), (3) lectin‐Alexa568 excitation at 561 nm (yellow), emission 580–620 nm (red), (4) chlorophyll A autofluorescence excitation 633 nm (red), emission 650–720 nm (far red). As a consequence, the microalgae will show up in the far red channel. If the sample harbors cyanobacteria that contain chlorophyll A and phycobilins, the overlay of the red and far red channels results in the signal of the cyanobacteria. Be aware that the overlay color depends on what false colors are allocated to each channel. This might be handled in different ways depending on what lab philosophy is established.

The colors mentioned in the above sections are true colors of the visible spectrum. However as the photomultiplier of the instrument “sees” only gray values, false colors have to be allocated to each channel. The monitor shows color in the red‐green‐blue (RGB) mode. In our definition of channel colors we decided for: reflection = white/gray, visible green in microscope = green, visible orange/red in microscope = red, the remaining color for the far red part of the spectrum = blue. Of note: The true visible green or orange or red color tone seen by eye in the microscope is not identical with the artificial standard RGB‐green or RGB‐red allocated by the user on the monitor.

Stains for UV laser (364 nm) or 405 nm laser diode—a popular nucleic acid stain is 4′,6‐Diamidin‐2‐phenylindol (DAPI) which ideally requires UV excitation. A critical point is the very broad emission spectrum of DAPI. In many CLSMs a much cheaper 405 nm laser diode is available as a replacement. However, this laser line is at the edge of the excitation spectrum of DAPI and, as a result, the emission signal is not that strong. The same is true for Calcofluor White‐M2R which is used for staining fungal cell walls or β 1 → 3, β 1 → 4 polysaccharides in the biofilm matrix.

Bio‐physico‐chemical properties—there are several enzyme kits offered, a very suitable one for laser microscopy is measuring the phosphatase activity. The activity becomes visible due to a fluorescent precipitate at the location of the enzyme (van Aarle et al. 2001). Redox sensors are quite useful for measuring oxidative stress of microbial communities, for example, with CellROX. Another redox sensor (BacLight RedoxSensor) measures the activity of bacterial reductase, indicating changes in the electron transport chain. The porosity of biofilms can be examined using fluorescently labeled dextrans or with fluorescent beads having different sizes and surface characteristics (Lawrence, Swerhone, et al. 2016). A relatively new approach for sensing the local biofilm habitat is using core shell silica nanoparticles. They contain two fluorochromes, one as a reference for their location and another one as a sensor dye. This so‐called C‐dots were applied to analyze the pH in microbial biofilms (Hidalgo et al. 2009).

Fluorescence in situ hybridisation (FISH)—in the meantime, there are many variations of the original FISH procedure published. However, FISH will not be treated in this paper as there is a huge record of original literature available with all the details. Ideally, FISH should be applied if the probes can be designed in house in order to make sure that the binding specificity is correct, that the protocol is tightly followed, and that all the necessary controls can be done. The reader should be also aware that FISH can be easily applied to defined mixed cultures and in activated sludge samples. However, it will be rather difficult if dense communities, environmental samples, or extreme habitats have to be FISHed. For more information, see the table at the end of the chapter with some useful literature to get started.

Other staining techniques—there are many additional options for staining, depending on the sample properties and the expected prokaryotic and eukaryotic microorganisms (bacteria, archaea, cyanobacteria, microalgae, fungi, and protists). For example, there are several stains for eukaryotic cell organelles (fungi and protists). For more information see “The Molecular Probes Handbook.”

## 8 CLSM Practical

### 8.1 How to Start

Before starting with laser microscopy, you have to switch on the CLSM for stabilizing the whole setup. Furthermore, especially gas lasers need to warm up (60 min) in order to have a stable power output. At the same time, you switch on the lamp of the epifluorescence microscope attached to the CLSM. You may also start up the computer and load the software of the microscopy company controlling the instrument.

At the beginning, you should also get familiar with the particular company software driving the CLSM. Usually there is a window or monitor showing the interface with all the settings and a second window or monitor showing the actual image. In addition, make sure that you understand the functionality of the control panel (if available) meaning what function is set on which knob for handling the instrument properly. Usually the operator uses both the mouse as well as the control panel simultaneously, together with the keyboard.

It's a good idea to use first an artificial sample, for example, with fluorescent beads to get familiar with all the software functions, the scan routine, and the effect on the resulting image. These beads have a defined dimension and measure for excitation and emission, and as there are many of them, there are enough available if some of them have been bleached. The beads are available in suspension or readily mounted on a microscope slide. In a second step, you may use a biological sample stained with a single fluorochrome in order to experience the difference between epifluorescence and confocal mode and the resulting image dataset.

### 8.2 Pre‐Settings

As a next step, you should define a number of pre‐settings. This may be, for example, the format in pixels, which for a square image might be 1024 × 1024, but other formats are possible. As another setting, the bit depth of the data has to be defined, resulting in the following pixel intensities: 8 bit—256 gray values, 12 bit—4096 gray values (scientific standard). If using 12 bit, make sure that subsequent image analysis software can handle the data. You may also double‐check the settings for scan speed (mostly medium or standard), scan direction (uni‐ or bi‐directional), pinhole (usually = 1), zoom (at start = 1), and average (none in the first place). After equilibration of the lasers, some of them are set in a coarse way to a low power output directly at the laser to prevent bleaching of the sample. Further adjustment in the software enables fine‐tuning of this parameter. The aim is to lower the laser power as much as possible and, at the same time, get enough emission signal for imaging.

### 8.3 Filter and Channel Settings

**FIGURE 11:**
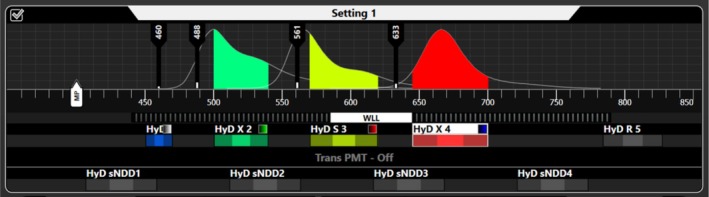
Example of a CLSM screenshot section showing a 4‐channel setting. In the upper area the four excitation lines of the white laser at 460, 488, 551, and 633 nm. In the lower area the width of four detection channels indicated in the color of the visible spectrum, blue—reflection (at the point of excitation), green—for example, FITC, Syto9, or Alexa488, yellow/orange—for example, TRITC, Alexa561 or phycobilins (cyanobacteria), red—for example, Alexa647 or chlorophyll A (cyanobacteria and algae). The color square top right of the detection channels indicates the false color allocation (gray—reflection channel, green—for the green emission, red for the orange/red emission, blue—for the far red emission). Please notice the emission spectra in the upper area of three standard fluorochromes indicating potential channel crosstalk. From Leica Microsystems.

Then you may define the filter settings to be used for a particular sample and staining. In other words, you define the beam path from the laser via the scan head and sample to the detector (Figure 11). This includes settings for filters (excitation/emission) and beam splitters as well as the number of detectors (emission channels). In order to set the beam pass correctly, you need to know the absorption and emission spectrum of your fluorochrome (look up in the internet) and the possible autofluorescence of your sample. The question arises: where in the spectrum are sample emissions (autofluorescence) and whether there is one or several “free windows” for applying a particular fluorochrome. Again refer to the websites called “spectra viewer,” where you can look up laser lines in combination with fluorochrome excitation and emission spectra in order to define the optimal settings for a particular sample and staining (see Table at the end).

### 8.4 Signal to Noise Ratio

**FIGURE 12:**
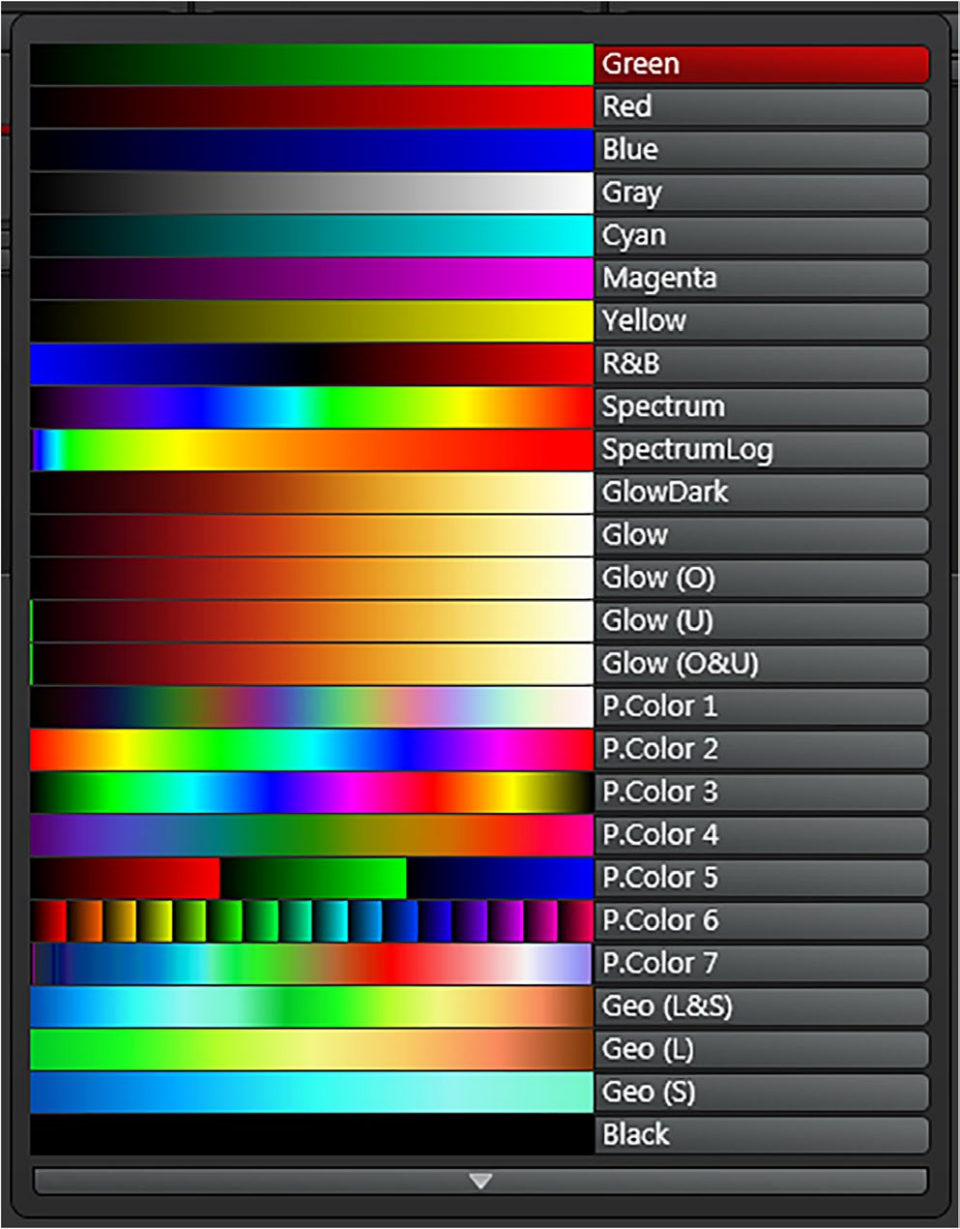
Lookup tables for RGB false color allocation. For example Green, Red, Blue for three channel imaging. Unfortunately the lookup table for Glow over‐under (Glow O&U) shows only the green bar for zero pixel intensity (left) but not the blue maximum pixel intensities (right). From Leica Microsystems.

After visual examination of the biofilm sample in the brightfield or epifluorescence mode and selecting an area of interest with the optimal lens, you switch the instrument into the laser mode. Next is optimization of the detector settings with respect to signal and contrast. For this purpose, there are different lookup tables available from different companies. A prominent lookup table for this purpose is called “glow over under” (GOU). As an example, for a Leica instrument the GOU shows for 8 bit data the pixel intensity = 0 in green color, the pixel intensity 255 in blue color and the intermediate pixel intensities in different shades of orange (Figure 12). For a Zeiss instrument the GOU shows for 8 bit data the pixel intensity = 0 in blue color, the pixel intensity 255 in red color and the intermediate pixel intensities in different shades of gray. The perfect setting should be (for Leica) a green background with a few dark pixels to make sure the background signal is just above zero whereas at the maximum you should see only a few saturated blue pixels. By this means, the optimal signal to noise ratio allows to use the full dynamic range.

### 8.5 Defining a Volumetric Dataset

After optimizing the signal to noise ratio, recording a series of *XY* images in *Z* direction results in a 3D image data stack. There are three coordinates to consider. *XY* direction is pre‐determined by selecting a specific objective lens, thus you know the dimensions of your image in *X* and *Y* in μm. *Z* (axial) direction defined by the user requires determination of the start and end of the object of interest. This setting is a bit tricky due to the elongation of confocal signals in the axial (*Z*) direction (see explanation of point spread function). By defining the start (biofilm surface) and end (biofilm at substratum), a first important number shows up, the thickness of the sample in μm. This number will determine the number of optical sections to be collected. Ideally, the step size should be in the range of the pixel size, resulting in a cube. But often, for example, due to bleaching, it has to be several times the pixel size. For example, if the biofilm is 25 μm thick, based on the size of a bacterium with about 1 μm, sectioning of the sample at every 1 μm or 0.5 μm makes sense. However, be aware that this optical sectioning may not match the Nyquist criterion (see below).

### 8.6 Bleaching

There are several reasons for bleaching ‐ Averaging. Some of them are caused by wrong microscope settings; others are induced by the user. The main reasons are listed in Table 3.

**TABLE 3:** Reasons for bleaching of fluorochromes.

Reasons for bleaching	Comments
Visual observation takes too long	Responsibility of the biologist
The fluorochrome is not stable	Some organic fluorochromes
The laser power is set to high	The laser should be set to a value which results in an acceptable signal to noise ratio
Defining the parameters takes too long	Especially with beginners
The pixel resolution is too high	Defined by the operator
The step size is too small	If fulfilling Nyquist criteria
A zoom factor is used	A zoom factor should only be used to match optical and digital resolution
The pixel time is too high	Low scan speed
Averaging	Averaging (bleaching) versus deconvolution (no bleaching)

If all other bleaching reasons are controlled, the next decision is about averaging, yes or no. By using an average setting, lowering the noise in an image is possible. However, averaging means more bleaching, consequently deconvolution maybe the better choice for improved signal to noise ratio and thus resolution. For doing deconvolution properly, recording of data must be at the Nyquist rate and the PSF of the objective lens has to be measured using tiny fluorescent beads (100 nm). Ideally, this measurement should be done within the sample at different height levels. As the measurement of the PSF is time‐consuming and Nyquist means bleaching, deconvolution is not frequently applied in microbiology. Finally, there is a need for a compromise in order to get improved datasets.

### 8.7 Recording and Sampling Data

Another issue arises in terms of recording and sampling. There are several reasons for recording CLSM data sets: recording the perfect image, analyzing an experiment with multiple samples, collecting data for semi‐quantification, and consideration of settings according to Nyquist for subsequent deconvolution. Consequently, there is not one single approach or setting that gives the best result. The one perfect 2D image or 3D dataset often aimed at is only possible if the sample is mounted and stained in the best possible way. In addition, it requires “playing” with settings and having plenty of time, thereby recording multiple datasets of the same content at the highest quality. In contrast, if many samples from an experimental series have to be analyzed, usually a certain time slot is available, and thus recording the single datasets in a short time frame is required. For semi‐quantitative results, imaging of replicate samples at many locations is necessary. At this point, the sampling area in μm^2^ becomes important in order to satisfy statistical requirements (Korber et al. 1992; Venugopalan et al. 2005). With respect to biofilms, the different numbers published show the need for taking as many datasets as possible in the available timeframe.

### 8.8 Triangle of Frustration

**FIGURE 13:**
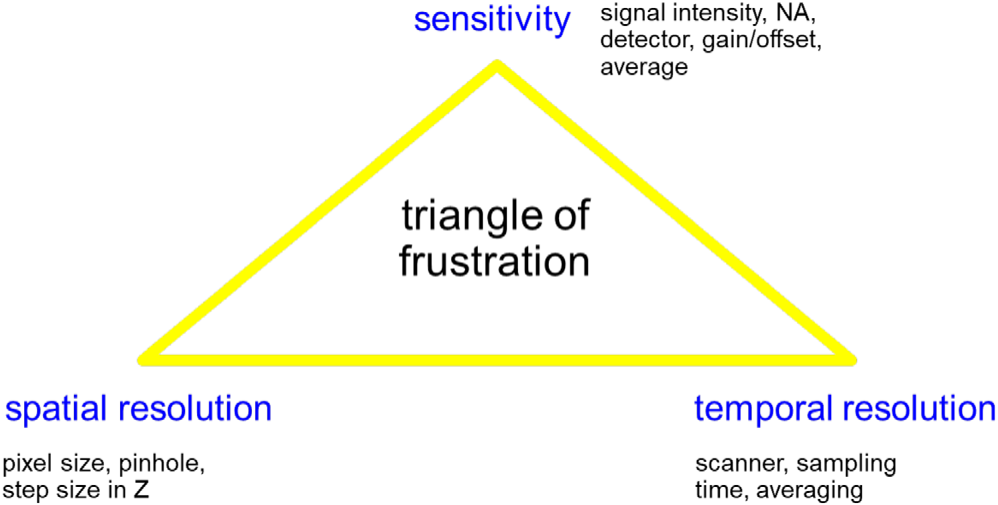
Important parameters that have to be adjusted with a compromise according to the requirements of a specific application. Designed according to www.zmb.unizh.ch.

In order to decide in what way a confocal dataset needs to be recorded, certain aspects have to be considered: the spatial resolution, the temporal resolution, and the sensitivity. As not all of these parameters can be perfectly optimized simultaneously, a triangle of frustration has been proposed (Figure 13).

As a result, the question has to be answered: What is the “Must Have” in a designed experiment? Most settings have their advantages but also limitations, and consequently, compromises are inevitable. Finally, it comes down to the main request of a particular CLSM application.

### 8.9 Acquisition Protocol

Over many years of CLSM, it turned out that a protocol in spreadsheet format is extremely helpful. This protocol contains all the important sample details, mounting and staining, together with the major microscope parameters (Table 4). In addition, the datasets are numbered in a meaningful way, for example, initials of user, then numbering from 001 plus a code for the channels, as the company file format may not show the details of the data file (e.g., number of channels). Although the datasets recorded and saved in the companies file format also contain all of the numerous instrument parameters, their lookup requires the microscope software. Thus, the spreadsheet file with only the most important parameters is very useful if you go back and consider what has been done weeks or months ago. On top of that, the spreadsheet protocol should contain a section called “comments” in which all the details of the individual images discussed during the recording session are written down. These “comments” are extremely helpful for later understanding, judgment, and selection of image data.

**TABLE 4:** Spreadsheet proposal as a protocol for CLSM work containing the most important parameters that are later needed for writing up reports, posters, thesis, and publications.

Title in spreadsheet	Explanation
Name	Person using the laser microscope
Title	Of the project, experiment
Sample	Name, number, type, and mounting
Stain	Fluorochrome and probes applied
File	Name of file saved (e.g., initials of user, number from 001 to …, letter indicating channels)
Method	Filter combination used for imaging (e.g., FITC‐TRITC‐CY5)
Scan mode	*XY* in *Z* direction, *XZ* in *Y* direction
Format	1024 × 1024 or 2048 × 2048 or other
Speed	Medium or fast, or value number in hertz
Lens	Objective lens magnification, numerical aperture, immersion (water, glycerol, oil), water‐immersible (dipping)
Pinhole	Airy = 1 or other (smaller = less intensity, higher resolution, larger = brighter signal, lower resolution)
Zoom	Zoom factor used (see matching of optical and digital resolution)
Image size	Field of view in micrometer
Pixel size	In micrometer Note 1: it will change if a zoom factor is used! Note 2: this will be needed if later in Photoshop a scale bar is added!
Thickness	In micrometer, for example, biofilm thickness
Step size	Defined by the user
Sections	Number of optical sections calculated by the software
Average	Type and value number of averaging
Laser line 1, 2, 3, …	Laser line(s) used for scanning a sample. (e.g., 488 for FITC, 561 for TRITC and 633 for CY5)
Emission range 1, 2, 3, …	Depending on CLSM brand, glass filter(s) used (e.g., band pass filter, long pass filter, …) or nanometer range if other detection (e.g., 500–550 nm for FITC, 575–610 nm for TRIC and 650–700 nm for CY5)
Gain PMT 1, 2, 3, …	Number to read out in software or control panel, important to judge emission signal intensity (staining quality) and possible noise
Other	Specific notes
Comments	**Most important!** Write down everything discussed during CLSM in order to remember later what was done, why it was done this way, what is seen in the data set, how it should be looked at and projected later, …

*Note:* A downloadable file is supplied in the supplement (Microsoft Excel).

### 8.10 Saving the Dataset

**FIGURE 14:**
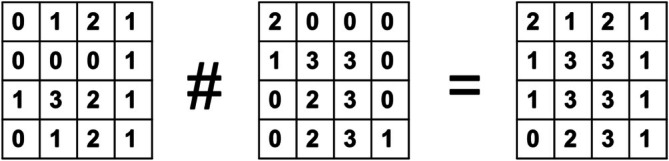
Calculation of maximum intensity projection (MIP) using a simple digital image with 4 × 4 low intensity pixels and two optical sections only. In the resulting MIP, the highest pixel intensity is projected (right).

Apart from the original dataset and the spreadsheet protocol, it is a good idea to save a maximum intensity projection (MIP) in a separate folder. The MIP is calculated by running through the single 2D images at each pixel location in the axial direction and looking for the highest pixel value, which finally is projected as a 2D image (Figure 14). Depending on the image content and pixel intensities, a MIP with fewer and less intense pixels looks very acceptable, even for publishing, whereas a MIP with many pixels at high intensities looks overloaded and very bright. However, that does not mean the dataset is not useful, but it must be projected in another way. In any case, the MIP represents not the final result of CLSM but an intermediate result as a two‐dimensional image, which may serve as a basis for later discussion. As one cannot memorize the many optical sections of a single three‐dimensional dataset, the MIP also becomes finally the image to remember. Both the MIP and the spreadsheet file are small files, easily saved on everybody's laptop for quick access. For image analysis of the very large original multichannel 3D dataset saved on a server, usually a high‐end PC and additional programs are needed, which can load the file header of the individual microscopy companies with all its machine parameters.

## 9 Digital Images and Resolution

### 9.1 Digital Data Structure

Old‐fashioned cameras took analogue (continuous) images (photos) on a film. Digital images as recorded by CLSM are pixelated images (data) saved at a defined resolution, for example, 1024 × 1024 pixels (picture elements). As a result, the single 2‐dimensional image on the monitor has a pixel resolution in *XY*, an image data stack (three‐dimensional) has a series of *XY* images in the Z direction, whereas a multichannel data set has multiple three‐dimensional data stacks. Behind each pixel stands a gray value for signal intensity at this specific location. This gray value codes for 8‐bit data 256 and for 12‐bit data 4096 (scientific standard) pixel intensities. On top of that, the pixels from different emission channels, although gray values only, code for a particular user‐defined color in the emission range. The colors on a monitor shown in red‐green‐blue (RGB) mode are called an additive color model. In contrast, the colors used for printing are cyan‐magenta‐yellow‐key (CMYK) represent a subtractive color model.

Remember—The three‐dimensional data sets are built of a series of two‐dimensional images at a defined step size. The *X*Y dimension is determined by the lens and the zoom factor, whereas the height (step size) is defined by the user. Consequently, the 3D digital data sets are assembled of volume elements (voxels). It is important to note that in many cases the voxel is much larger in the *Z* direction if compared to the resolution in the XY direction. This means the voxel looks like a long cuboid.

### 9.2 Digital Versus Microscope Resolution

**FIGURE 15:**
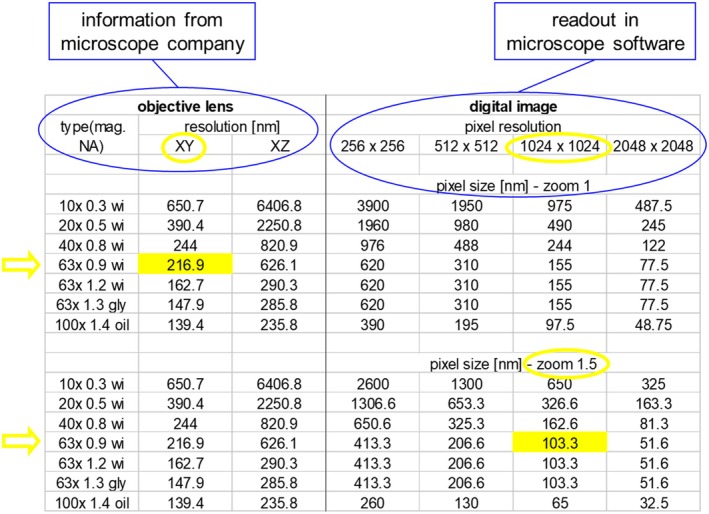
Example for calculating the proper match between optical resolution and pixel resolution. If the lens “63× NA 0.9 wi” has a resolution of 216.9 nm (left), this value is multiplied by 1.4 and divided by 3 resulting in 101.2. Now by searching the pixel resolution versus the zoom factor (right), a value is selected which comes close to 101.2. In this example it would be for the lens “63× NA 0.9 wi” at a pixel resolution of 1024 × 1024 and a zoom factor of 1.5.

For the best image results, matching the optical resolution of the microscope (lens) with the resolution of the image (pixels) is required. For this purpose, the optical resolution of the lens in *XY* (supplied by the microscope company for reflection) is multiplied by 1.4 (for fluorescence) and divided by 3, which results in the optimal pixel size in nm. Selecting the appropriate pixel resolution in combination with a matching zoom setting will allow the adjustment of the correct pixel size (Figure 15). Nevertheless, this may not always match the Nyquist criteria.

## 10 LSM Cleanliness and Safety

When working at the microscope, the major optical components, the objective lenses, have to be clean and free of scratches. Cleanliness is important due to the various immersion lenses (water, glycerol, and oil) frequently used for CLSM. Please refer to the recommendation of the supplier for cleaning tissues and liquids. The issue of scratches becomes critical when using water immersible (dipping) lenses, especially if examination of biofilms on solid surfaces like rocks or pebbles is necessary. Due to a fully automated microscope stand with motors moving the stage as well as the revolver with the objective lenses, this becomes a real challenge. An easy way of testing the objective lens is using a binocular, which will show possible problems of the front lens.

Working with fluorochromes requires some considerations in terms of safety and health. For some fluorochromes, their toxicity and carcinogenicity are known (e.g., DAPI) but for most of the fluorochromes, their detailed toxicity is unknown. Thus, be careful in handling fluorochromes! As fluorochromes bind to specific biochemical constituents of the microbiological sample, they certainly bind to similar constituents of the user too. It is also a good idea, in fact a laboratory regulation, to collect fluorochrome liquid waste and fluorochrome‐contaminated lab disposals separately.

With respect to laser safety, there is hardly an issue, as the laser encased in the instrument does not pose any danger. In addition, during the scan a shutter will close the light pass to the ocular. Further, when the laser is scanning, the user's eyes usually focus on the software and monitor. The only “do‐not” criteria is manipulating the sample with a reflecting tool during the scan.

## 11 Quality Control and Performance

For testing the CLSM capability, there are several items available such as prepared slides with fluorescent beads of various sizes and colors as well as beads having a shell with a different color. In addition, there are slides with an imprinted geometric micro‐ and nano‐pattern that are useful for checking the quality of imaging. Very recently, fluorescent nano‐rulers based on the DNA origami technique became available. See the table at the end of the chapter with suppliers and products.

There is also a contradiction in terms of the instrument quality (company prestige), instrument functionality (trust in company technician) and the performance, usually confirmed by producing a colorful image (company demonstration). What was missing were simple tools for independently testing the performance of a CLSM instrument. This issue tackled by Zucker et al. revealed a number of tests easily done by the user (Zucker 2006). These tests not only allow a better understanding of the CLSM instrument, they are also helpful to explain to the technician of the microscope company what the technical problem is if something comes up. The various Zucker tests are explained in Table 5.

**TABLE 5:** Simple performance tests for functionality of the CLSM according to Zucker (Zucker 2006).

Performance test	Parameter examined
Microscope stage	The stability of the microscope stage is measured using the reflection of a test grid, the result should be the grid pattern without shift in *XY* or *Z*
Laser power	The laser power output is measured with a laser power meter at the objective lens, should be done every half year/annually
Laser stability	The laser intensity is measured over time for each laser line, the result should be a horizontal line
Galvanometer	For confirmation of a smooth scan a reflective test grid is scanned at different zoom settings, the result should show clear lines
Field illumination	With a fluorescent plastic slide or a fluorescent solution the equal illumination across the image is measured
Axial resolution	Measurement of the full width‐half maximum (FWHM) distance = resolution in axial direction using reflection
Dichroic filters, beam splitter	The different beam splitters are tested with respect to signal intensity using an image and calculating the mean gray scale value
Spectral scanning	Using a lamp with known spectrum, a lambda scan is done and compared with the given spectrum of the lamp
Spectral registration	Test beads with different fluorochromes inside and in the shell are measured and compared with the information from the supplier
Coefficient of variation	General performance parameter measured with test beads to detect noise

In microscopy facilities the quality control covers a number of tasks which should be done on a regular schedule. They include: inspection and cleaning, illumination path, channel alignment, resolution, scanner, detection path and stage. Very recently, these quality issues were the basis of an initiative called “Quality Assessment and Reproducibility for Instruments & Images in Light Microscopy” (QUAREP‐LiMi) (Nelson et al. 2021). In 14 working groups, each with a specific topic, the goal is to establish common quality standards, guidelines, metadata models, tools and protocols in order to improve the overall image quality and reproducibility (https://quarep.org/).

## 12 Laser Scanning Microscopy Variations

### 12.1 Photon Versus 2‐Photon

**FIGURE 16:**
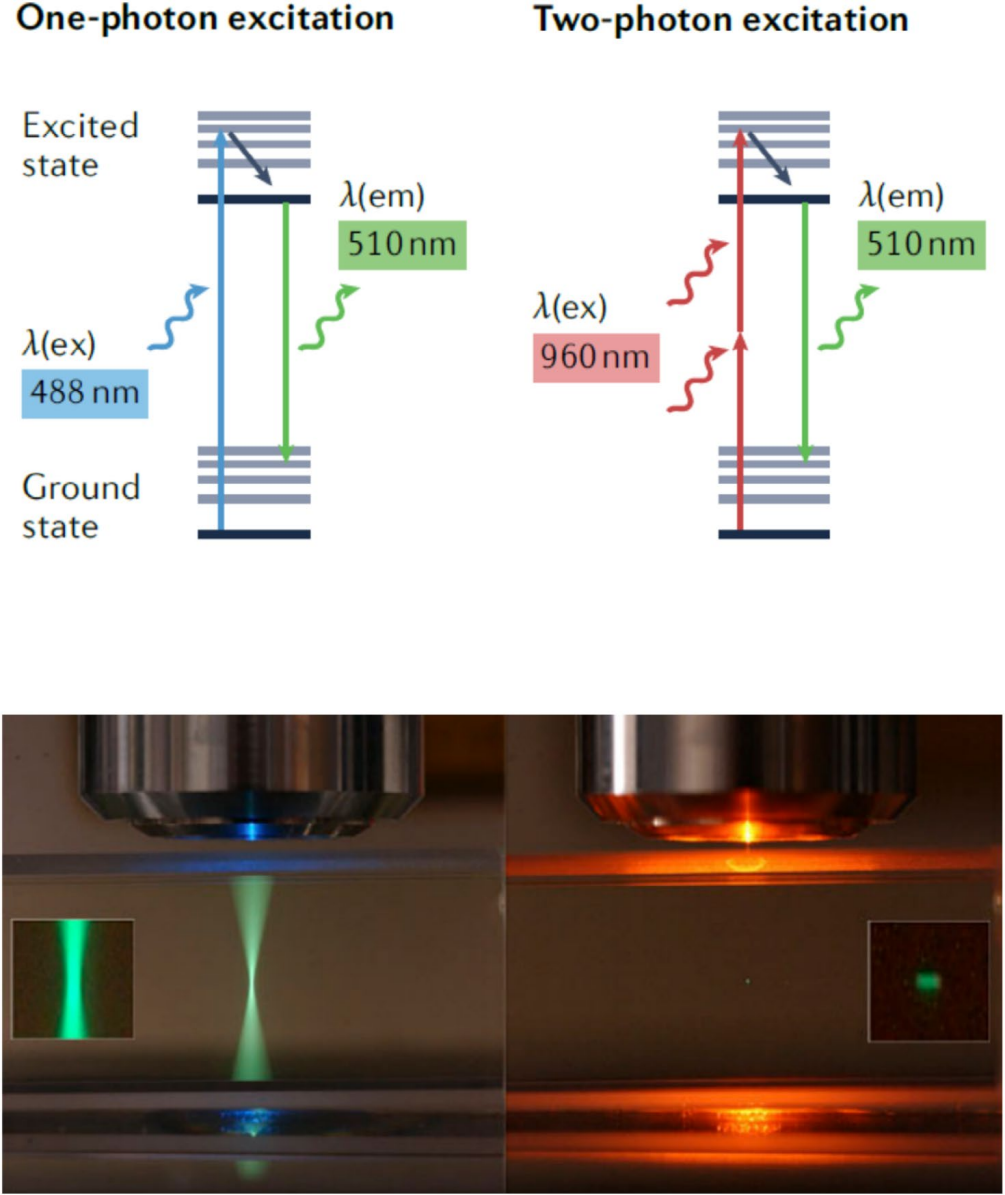
Comparison of one‐photon (confocal) and two‐photon excitation. Top: Please notice that the emission signal of the fluorochrome in both cases is in the identical range. From Scheele et al. (2022). Bottom: Area of laser illumination in one‐photon compared to two‐photon excitation. From https://blog.biodock.ai/one‐vs‐two‐photon‐microscopy/.

In traditional CLSM, the excitation of a fluorochrome achieved with high‐energy light requires one single photon. For this purpose, the various lasers available produce light from the UV, blue, green, orange, and red parts of the electromagnetic spectrum. Due to the shorter wavelengths, the laser light is scattered within the sample, especially in environmental biofilms, thereby limiting the depth of penetration and resolution. This limitation is circumvented by using a pulsed infrared laser for two‐photon excitation, which allows deeper penetration into a scattering sample. As a consequence, the resolution in deeper locations is higher. Depending on the samples properties, the imaging depth can be increased from 2 to 3 fold. This is achieved with two low‐energy photons at about double the wavelength when compared to one‐photon excitation (Figure 16). In order to achieve a two‐photon process (excitation), the two photons have to arrive at the fluorochrome more or less simultaneously. Of note, the two‐photon effect occurs only in the focal plane, and as a consequence, there is no bleaching above or below the focal plane. Be aware that in two‐photon excitation, the emission signal is recorded on the left side of the excitation (shorter wave length) which is opposed to one‐photon excitation, where the emission signal is always on the right side (longer wave length). Two‐photon excitation results in a number of advantages but also some limitations, as listed in Table 6.

**TABLE 6:** Advantages and limitations of two‐photon laser scanning microscopy.

Advantage	Comments
Deeper penetration into scattering media	Due to the longer wave length of infrared light
Inherent depth resolution	Imaging in deeper locations is possible
Excitation of the sample in the focal region only	No pinhole is necessary
No out of focus bleaching and cell damage	But bleaching in the focal region
No background fluorescence	If out of focus, no light reaches the background
No UV laser, no UV optics needed	No UV photo damage
IR is more benign to living cells	Compared to UV light
Large disparity between excitation and emission	In contrast to one‐photon excitation with emission towards the longer wavelength on the right (Stoke shift), in two‐photon excitation the emission is on the left at the location of one‐photon emission
**Limitations**	**Comments**
Theoretical resolution is lower in comparison to one‐photon excitation	In reality this effect is very minor
Not suitable to IR absorbing samples	For example plastics
Additional expensive hardware needed	Budget issue
Bleaching in focal plane	This might be higher if compared to one‐photon excitation
Unexpected two‐photon effects	To be aware of

### 12.2 Fluorescence Techniques

There are a number of F‐techniques, thus one should be familiar with the acronyms FRIM, FRAP, FRET, and FLIM. Fluorescence ratio imaging (FRIM) takes advantage of the fluorochrome sensitivity for an analyte. FRIM, for example, allows measuring of the pH in biofilms or cells (Siegumfeldt et al. 1999). Fluorescence recovery after photobleaching (FRAP) records the penetration of unbleached fluorochromes into a bleached area. FRAP allows determining the diffusion coefficient in a sample (Waharte et al. 2010). Fluorescence resonance energy transfer (FRET) represents a technique where the transfer of energy from a donor to an acceptor molecule is measured. FRET facilitates distance measurements at the nm scale, thereby enlarging the resolution of the microscope (Centonze et al. 2003). Fluorescence lifetime imaging (FLIM) requires additional hardware and software. Next to the fluorescence emission intensity, the fluorescence lifetime constitutes the second piece of information derived from a fluorochrome. FLIM is based on the sensitivity of a fluorochrome to its micro/nano environment and, as a result, reveals additional information from a specific location (Loeppmann et al. 2023; Walczysko et al. 2008). Recently, the analysis of lifetime imaging data sets was strongly improved and simplified by implementing the so‐called phasor approach. By this graphical method, the image is analyzed at each pixel, and a histogram is produced showing groups of pixels with different decay profiles (Digman et al. 2008; Malacrida et al. 2021; Torno et al. 2013).

### 12.3 Light Sheet Microscopy

This relatively new optical geometry for imaging is called light sheet fluorescence microscopy or selective plane illumination microscopy (Power and Huisken 2017; Stelzer et al. 2021). The sample is illuminated with a thin laser sheet, and the resulting signals are collected via a perpendicularly mounted objective lens. The technique was developed for imaging larger samples in cell biology, such as small embryos or organs. The samples are usually embedded in a gel cylinder, which is turned for imaging the sample from all directions. The advantages are minimal exposure to light, less photo damage, improved signal to noise ratio, imaging at high frame rates, and recording images over a long time period. In the meantime, there are several different implementations of the technique; nevertheless, only a few of them have been applied for specific microbiological samples (Zhang et al. 2021).

## 13 High Resolution Laser Microscopy Techniques

### 13.1 Improved Resolution

Since a few years, traditional CLSM has developed into a number of techniques for improved resolution. To remember, the resolution of the CLSM is roughly half the wavelength used for excitation. Consequently, 500 nm excitation (blue light) means about 250 nm resolution.

One of the first techniques to improve the resolution of CLSM was structured illumination microscopy. Structured illumination using a light pattern at various angles allows extraction of high frequency information, which finally results in double resolution (Gustafsson 2000).

Another option to increase the resolution is image restoration also called deconvolution. This treatment of image data sets was usually done after recording the images. However, in the meantime deconvolution “on the fly” is available. Thus during image recording the datasets are immediately deconvolved which results in highly resolved images (120 nm).

A hardware approach called image scanning microscopy takes advantage of a detector array instead of a point detector as in CLSM. As a straightforward technique, several companies have integrated this principle already into their microscopes using different approaches. The Airyscan microscope takes advantage of a microlens detector array with 32 elements. Each detector element acts as a small virtual pinhole with positioning information, resulting in a 1.7‐fold improved resolution and a 4–8× increase in signal to noise ratio (Korobchevskaya et al. 2017). The Re‐Scan technique employs two scanners, a confocal scanning unit in combination with a re‐scanning unit and detection by an EMCCD. As a result, the resolution is improved with an open pinhole by a factor of 1.4 (De Luca et al. 2017). A further version implemented into a spinning disc microscope uses a microlens array disk for excitation as well as a microlens pinhole array for detection. This combination, together with structured illumination, allows for 120 nm resolution (Hayashi and Okada 2015). Image scanning microscopy results in double lateral resolution (120–170 nm) and four times more contrast. In the near future, image scanning microscopy may change the common confocal routine and may supplant CLSM.

### 13.2 Nanoscopy (Nobel Prize in Chemistry 2014)

Implementation of imaging at even higher resolution established with three different approaches revealed resolutions well across the diffraction barrier. However, there are some restrictions to their applications as the sample has to somehow adapt to the highly advanced technique which limits the general application of nanoscopy (Schermelleh et al. 2010). In localisation or blink microscopy, switching individual fluorochromes on and off over time, calculating their precise location and final projection in a pointillism way facilitates a resolution into the range of 20 to 30 nm. This nanoscopy technique was developed by several groups creating many different acronyms and variations of the procedure (Lelek et al. 2021). Stimulated emission depletion (STED) microscopy uses one laser producing a confocal spot and a second laser with a donut‐shaped beam to deplete this spot in the outer region. By this means, achieving a resolution of 80 nm was feasible with the early setups (Hell 2007). In the meantime, several improved variations developed demonstrated resolutions down to 10 nm. The most recent improvements employ a new and fundamentally different localisation concept of a fluorochrome. One technique is called MinFlux, a combination of localisation microscopy and STED microscopy with a resolution of 1–3 nm. In this technique, the switchable fluorochromes are imaged similar to localisation microscopy. However, the localisation is achieved by a moveable donut‐shaped beam in an iterative way (Balzarotti et al. 2017). Another similar approach is called MinSTED where the fluorochromes are randomly switched on by a photochemical process. Then a circulating excitation donut localizes the fluorochrome and at the same time keeps the background low (Weber et al. 2021). In both approaches, the localisation is accomplished with the laser beam and not via the fluorochromes. In addition, they require fewer photons for imaging and allow 1 nm resolution in three dimensions. An overview of the current super resolution techniques has been published recently discussing the available commercial instruments (Mezache and Leterrier 2025).

## 14 Further Reading and Information—Books, Book Chapters, Reviews, Articles, Websites

Finally, to further access the broad field of biofilm CLSM and an in‐depth understanding of different theoretical and practical aspects, a selection of major sources for more detailed information is provided. They comprise fluorescence, optics, microscopy, and digital imaging analysis as well as a range of microbiological applications, including practical issues. The following tables with literature are split up based on their source and contain the references as well as comments on their content. Several internet sites with helpful tutorials are listed for easy access from everywhere. Different types of spectra viewer for optimal fluorochrome excitation and emission settings as well as specific items useful for confocal microscopy in general are listed as well.

### 14.1 General Confocal Microscopy and Image Analysis—Books

**Table:** 

Title	References	Comments
Confocal Laser Scanning Microscopy	Sheppard and Shotton (1997)	Easy readable short introduction for biologists
Introduction to Confocal Fluorescence Microscopy	Müller (2002)	Short introduction with more physical background information
Biological Confocal Microscopy	Pawley (2006)	The “bible” of confocal microscopy, very technical, 1000 pages
Digital Microscopy	Sluder and Wolf (2013)	Very useful introduction to fluorescence microscopy, optics, fluorescence techniques, digital image analysis
Fluorescence microscopy	Kubitscheck (2017)	Introduction to microscopy and nanoscopy techniques
Imaging: A Laboratory Manual	Yuste (2011)	Manual style information on instrumentation, labelling, advanced microscopy, with focus on cell biology, 1000 pages
Handbook of Fluorescent Probes and Research Chemicals	Haugland (2005)	Molecular Probes supplier, major source of fluorochromes with applications explained, 1000 pages
The Image Processing Handbook	Russ and Neal (2016)	Basics of general image processing with numerous visual examples, 1000 pages

### 14.2 Imaging in Microbiology—Books

**Table:** 

Title	References	Comments
Microbial Imaging	Savidge and Pothoulakis (2004)	Overview on different techniques and applications
Digital Image Analysis of Microbes	Wilkinson and Shut (1998)	Basics and various applications

### 14.3 Imaging of Biofilms by LSM—Reviews/Chapters

**Table:** 

Title	References	Comments
Confocal laser scanning microscopy and digital image analysis in microbial ecology	Caldwell et al. (1992)	First overview on CLSM applications
One‐photon versus two‐photon laser scanning microscopy and digital image analysis of microbial biofilms	Neu and Lawrence (2004)	Comparison of confocal and multi‐photon LSM
Confocal microscopy of biofilms—spatiotemporal approaches.	Palmer et al. (2006)	Flow cells, fluorescence techniques, CLSM, two‐photon LSM
Laser scanning microscopy	Lawrence and Neu (2007a)	Manual style, CLSM for examination of microbial communities
Laser scanning microscopy for microbial flocs and particles	Lawrence and Neu (2007b)	CLSM imaging techniques, quantitative imaging, applications
Advanced imaging techniques for assessment of structure, composition and function in biofilm systems	Neu et al. (2010)	Covers the following techniques: CLSM, MRI, and STXM
Advanced techniques for the in situ analysis of the of the biofilm matrix (structure, composition, dynamics) by means of laser microscopy	Neu and Lawrence (2014a)	Compilation of methods for studying the extracellular matrix
Investigation of microbial biofilm structure	Neu and Lawrence (2014b)	CLSM basics, probes and applications
Aquatic biofilms: Development, cultivation, analyses, and applications	Lawrence, Neu, et al. (2016)	Manual style, basics on aquatic biofilms and their investigation
Examination of microbial communities on hydrocarbons by means of laser scanning microscopy	Neu and Lawrence (2016a)	CLSM of microbes on solid, viscous and liquid hydrocarbon interfaces
Laser microscopy for the study of biofilms—Issues and options	Neu and Lawrence (2016b)	Focus on aquatic biofilms and bio‐aggregates

### 14.4 Microscopy Tutorials and Teaching Websites of Companies

**Table:** 

www	Company
https://www.leica‐microsystems.com/science‐lab/science‐lab‐home/	Leica
https://www.microscopyu.com/	Nikon
https://zeiss‐campus.magnet.fsu.edu/	Zeiss
https://svi.nl/Huygens‐Imaging‐Academy	SVI
https://www.thermofisher.com/de/de/home/brands/molecular‐probes.html	Invitrogen, Molecular Probes
https://www.thermofisher.com/de/de/home/life‐science/cell‐analysis/cell‐analysis‐learning‐center/molecular‐probes‐school‐of‐fluorescence.html	Invitrogen, Molecular Probes

### 14.5 Supply of Fluorescence Chemicals

**Table:** 

www	Details
https://www.thermofisher.com/de/de/home/life‐science/cell‐analysis/cellular‐imaging/fluorescence‐microscopy‐and‐immunofluorescence‐if.html	Specific fluorochromes, fluorochrome labels, and probe labelling kits
http://www.biostatus.com/	Specific fluorochromes
https://biotium.com/	Specific fluorochromes and probe labelling kits
https://www.tocris.com/product‐type/fluorescence‐imaging	Specific fluorochromes
https://www.ebbabiotech.com/	Specific fluorochromes
https://www.atto‐tec.com/	Fluorochrome labels
https://de.lumiprobe.com/	Fluorochrome labels
https://dyomics.com/en/	Fluorochrome labels
https://abberior.rocks/dyes‐and‐labels/	Fluorochrome labels

### 14.6 Publications on FISH

**Table:** 

Content	References
Introduction to FISH	Almeida and Azevedo (2021)
Critical review on EUB338 probe	Bouvier and Giorgio (2003)
FISH application	Bottari et al. (2006)
New FISH variations	Zwirglmaier (2005)
Multi‐color FISH	Lukumbuzya et al. (2019)
Review on improved FISH	Amann and Fuchs (2008)
FISH glossary, several suitable for microbes	Volpi and Bridger (2008)

### 14.7 Spectra Viewer Websites

**Table:** 

https://www.thermofisher.com/order/fluorescence‐spectraviewer#!/
https://www.bdbiosciences.com/en‐de/resources/bd‐spectrum‐viewer
https://www.chroma.com/spectra‐viewer
https://searchlight.semrock.com/
https://www.novusbio.com/spectraviewer
https://www.aatbio.com/fluorescence‐excitation‐emission‐spectrum‐graph‐viewer
https://www.biolegend.com/spectraanalyzer

### 14.8 Other Helpful Items and Sources

**Table:** 

www	Material
https://www.marienfeld‐superior.com/precision‐cover‐glasses‐thickness‐no‐1‐5h‐tol‐5‐m.html	High quality coverslips
https://gracebio.com/	Coverwell imaging chambers for upright microscopes
https://ibidi.com/	Chambered coverslips for inverted microscopes
https://www.thermofisher.com/	Fluorescent beads and test slides
https://argolight.com/	Test slides with different geometric and fluorescence patterns including 3d
https://www.gattaquant.com/de	Nano‐rulers for confocal microscopy

## Author Contributions

**Thomas R. Neu:** writing – original draft, conceptualization, writing – review and editing, project administration. **Ute Kuhlicke:** methodology, validation, investigation, writing – review and editing.

## Conflicts of Interest

The authors declare no conflicts of interest.

## Supporting information


**Data S1:** Supplementary Tables.

## Data Availability

Data sharing is not applicable to this article as no new data were created or analyzed in this study.

## References

[jemt70064-bib-0064] Almeida, C. , and N. F. Azevedo . 2021. “An Introduction to Fluorescence In Situ Hybridization in Microorganisms.” Fluorescence In‐Situ Hybridization (FISH) for Microbial Cells: 1–15. 10.1007/978-1-0716-1115-9_1. 33576979

[jemt70064-bib-0001] Amann, R. , and B. M. Fuchs . 2008. “Single‐Cell Identification in Microbial Communities by Improved Fluorescence in Situ Hybridisation.” Nature Reviews Microbiology 6: 339–348. 18414500 10.1038/nrmicro1888

[jemt70064-bib-0002] Balzarotti, F. , Y. Eilers , K. C. Gwosch , et al. 2017. “Nanometer Resolution Imaging and Tracking of Fluorescent Molecules With Minimal Photon Fluxes.” Science 355, no. 6325: 606–612. 28008086 10.1126/science.aak9913

[jemt70064-bib-0065] Bottari, B. , D. Ercolini , M. Gatti , and E. Neviani . 2006. “Application of FISH Technology for Microbiological Analysis: Current State and Prospects.” Applied Microbiology and Biotechnology 73, no. 3: 485–494. 10.1007/s00253-006-0615-z. 17051413

[jemt70064-bib-0003] Bouvier, T. , and P. A. d. Giorgio . 2003. “Factors Influencing the Detection of Bacterial Cells Using Fluorescence in Situ Hybridization (FISH): A Quantitative Review of Published Reports.” FEMS Microbiology Ecology 44: 3–15. 19719646 10.1016/S0168-6496(02)00461-0

[jemt70064-bib-0004] Caldwell, D. E. , D. R. Korber , and J. R. Lawrence . 1992. “Confocal Laser Scanning Microscopy and Digital Image Analysis in Microbial Ecology.” Advances in Microbial Ecology 12: 1–67.

[jemt70064-bib-0005] Centonze, V. E. , M. Sun , A. Masuda , H. C. Gerritsen , and B. Herman . 2003. “Fluorescence resonance energy transfer imaging microscopy.” Methods in Enzymology 360: 542–560. 12622167 10.1016/s0076-6879(03)60127-8

[jemt70064-bib-0006] Christensen, B. B. , C. Sternberg , J. B. Anderson , et al. 1999. Methods in Enzymology. Academic Press. 10.1016/s0076-6879(99)10004-1 10547780

[jemt70064-bib-0007] De Luca, G. , R. Breedijk , R. Hoebe , S. Stallinga , and E. Manders . 2017. “Re‐Scan Confocal Microscopy (RCM) Improves the Resolution of Confocal Microscopy and Increases the Sensitivity.” Methods and Applications in Fluorescence 5, no. 1: 15002. 10.1088/2050-6120/5/1/015002 28120817

[jemt70064-bib-0008] Decho, A. W. , and T. Kawaguchi . 1999. “Confocal Imaging of in Situ Natural Microbial Communities and Their Extracellular Polymeric Secretions Using Nanoplast Resin.” BioTechniques 27: 1246–1252. 10631505

[jemt70064-bib-0009] Digman, M. A. , V. R. Caiolfa , M. Zamai , and E. Gratton . 2008. “The Phasor Approach to Fluorescence Lifetime Imaging Analysis.” Biophysical Journal 94, no. 2: L14–L16. 17981902 10.1529/biophysj.107.120154 PMC2157251

[jemt70064-bib-0010] Droppo, I. G. , D. T. Flannigan , G. G. Leppard , C. Jaskot , and S. N. Liss . 1996. “Floc stabilization for multiple microscopic techniques.” Applied and Environmental Microbiology 62: 3508–3515. 16535412 10.1128/aem.62.9.3508-3515.1996 PMC1388950

[jemt70064-bib-0011] Gräf, R. , J. Rietdorf , and T. Zimmermann . 2005. “Live Cell Spinning Disk Microscopy.” In Microscopy Techniques, edited by J. Rietdorf , 57–75. Springer Berlin Heidelberg. 10.1007/b102210 16080265

[jemt70064-bib-0012] Gustafsson, M. G. L. 2000. “Surpassing the Lateral Resolution Limit by a Factor of Two Using Structured Illumination Microscopy.” Journal of Microscopy 198: 82–87. 10810003 10.1046/j.1365-2818.2000.00710.x

[jemt70064-bib-0013] Haugland, R. P. 2005. Handbook of Fluorescent Probes and Research Chemicals. Molecular Probes.

[jemt70064-bib-0014] Hayashi, S. , and Y. Okada . 2015. “Ultrafast Superresolution Fluorescence Imaging With Spinning Disk Confocal Microscope Optics.” Molecular Biology of the Cell 26, no. 9: 1743–1751. 25717185 10.1091/mbc.E14-08-1287 PMC4436784

[jemt70064-bib-0015] Hell, S. W. 2007. “Far‐Field Optical Nanoscopy.” Science 316: 1153–1158. 17525330 10.1126/science.1137395

[jemt70064-bib-0016] Hidalgo, G. , A. Burns , E. Herz , et al. 2009. “Functional Tomographic Fluorescence Imaging of pH Microenvironments in Microbial Biofilms by Use of Silica Nanoparticle Sensors.” Applied and Environmental Microbiology 75: 7426–7435. 19801466 10.1128/AEM.01220-09 PMC2786433

[jemt70064-bib-0017] Jerome, W. G. , and R. L. Price . 2018. Basic Confocal Microscopy. Springer Nature.

[jemt70064-bib-0018] Korber, D. R. , J. R. Lawrence , M. J. Hendry , and D. E. Caldwell . 1992. “Programs for Determining Statistically Representative Areas of Microbial Biofilms.” Binary 4: 204–210.

[jemt70064-bib-0019] Korobchevskaya, K. , B. C. Lagerholm , H. Colin‐York , and M. Fritzsche . 2017. “Exploring the Potential of Airyscan Microscopy for Live Cell Imaging.” Photonics 4, no. 3: 41.

[jemt70064-bib-0020] Kubitscheck, U. 2017. Fluorescence Microscopy. Wiley‐Blackwell.

[jemt70064-bib-0021] Lawrence, J. R. , and T. R. Neu . 2007a. “Laser Scanning Microscopy.” In Methods for General and Molecular Microbiology, edited by C. A. Reddy , T. J. Beveridge , J. A. Breznak , G. A. Marzluf , T. M. Schmidt , and L. R. Snyder , 34–53. ASM.

[jemt70064-bib-0022] Lawrence, J. R. , and T. R. Neu . 2007b. “Laser Scanning Microscopy for Microbial Flocs and Particles.” In Environmental Colloids: Behavior, Structure and Characterisation IUPAC Series Volume 10, edited by K. J. Wilkinson and J. R. Lead , 469–505. John Wiley.

[jemt70064-bib-0023] Lawrence, J. R. , T. R. Neu , A. Paule , D. R. Korber , and G. M. Wolfaardt . 2016. “Aquatic Biofilms: Development, Cultivation, Analyses, and Applications.” In Manual of Environmental Microbiology, 4th ed. American Society of Microbiology.

[jemt70064-bib-0024] Lawrence, J. R. , G. D. W. Swerhone , U. Kuhlicke , and T. R. Neu . 2016. “In Situ Evidence for Metabolic and Chemical Microdomains in the Structured Polymer Matrix of Bacterial Microcolonies.” FEMS Microbiology Ecology 92, no. 11: fiw183. 10.1093/femsec/fiw183 27562775

[jemt70064-bib-0025] Lelek, M. , M. T. Gyparaki , G. Beliu , et al. 2021. “Single‐molecule localization microscopy.” Nature Reviews Methods Primers 1, no. 1: 39. 10.1038/s43586-021-00038-x PMC9160414 35663461

[jemt70064-bib-0026] Loeppmann, S. , J. Tegtmeier , Y. Shi , et al. 2023. “Using Fluorescence Lifetime Imaging to Disentangle Microbes From the Heterogeneous Soil Matrix.” Biology and Fertility of Soils 59, no. 2: 249–260.

[jemt70064-bib-0027] Lukumbuzya, M. , M. Schmid , P. Pjevac , and H. Daims . 2019. “A Multicolor Fluorescence in Situ Hybridization Approach Using an Extended Set of Fluorophores to Visualize Microorganisms.” Frontiers in Microbiology 10, no. 1383: 1383. 31275291 10.3389/fmicb.2019.01383 PMC6593226

[jemt70064-bib-0028] Malacrida, L. , S. Ranjit , D. M. Jameson , and E. Gratton . 2021. “The Phasor Plot: A Universal Circle to Advance Fluorescence Lifetime Analysis and Interpretation.” Annual Review of Biophysics 50, no. 1: 575–593. 10.1146/annurev-biophys-062920-063631 33957055

[jemt70064-bib-0029] Mezache, L. , and C. Leterrier . 2025. “Advancing Super‐Resolution Microscopy: Recent Innovations in Commercial Instruments.” Microscopy and Microanalysis 31, no. 2: ozaf004. 10.1093/mam/ozaf004 40183990

[jemt70064-bib-0030] Müller, M. 2002. Introduction to Confocal Fluorescence Microscopy. Shaker Publishing BV.

[jemt70064-bib-0031] Nelson, G. , U. Boehm , S. Bagley , et al. 2021. “QUAREP‐LiMi: A Community‐Driven Initiative to Establish Guidelines for Quality Assessment and Reproducibility for Instruments and Images in Light Microscopy.” Journal of Microscopy 284, no. 1: 56–73. 34214188 10.1111/jmi.13041 PMC10388377

[jemt70064-bib-0032] Neu, T. R. , and U. Kuhlicke . 2017. “Fluorescence Lectin Bar‐Coding of Glycoconjugates in the Extracellular Matrix of Biofilm and Bioaggregate Forming Microorganisms.” Microorganisms 5, no. 1: 5. 28208623 10.3390/microorganisms5010005 PMC5374382

[jemt70064-bib-0033] Neu, T. R. , and U. Kuhlicke . 2022. “Matrix Glycoconjugate Characterization in Multispecies Biofilms and Bioaggregates From the Environment by Means of Fluorescently‐Labeled Lectins.” Frontiers in Microbiology 13: 940280. 36003926 10.3389/fmicb.2022.940280 PMC9395170

[jemt70064-bib-0034] Neu, T. R. , and J. R. Lawrence . 2004. “One‐Photon Versus Two‐Photon Laser Scanning Microscopy and Digital Image Analysis of Microbial Biofilms.” Methods in Microbiology 34: 87–134.

[jemt70064-bib-0035] Neu, T. R. , and J. R. Lawrence . 2014a. “Advanced Techniques for in Situ Analysis of the Biofilm Matrix (Structure, Composition, Dynamics) by Means of Laser Scanning Microscopy.” In Microbial Biofilms—Methods and Protocols. Methods in Molecular Biology, edited by G. Donelli , 43–64. Springer. 10.1007/978-1-4939-0467-9_4 24664825

[jemt70064-bib-0036] Neu, T. R. , and J. R. Lawrence . 2014b. “Investigation of Microbial Biofilm Structure by Laser Scanning Microscopy.” Advances in Biochemical Engineering/Biotechnology 146: 1–51. 10.1007/10_2014_272 24840778

[jemt70064-bib-0037] Neu, T. R. , and J. R. Lawrence . 2016a. “Examination of Microbial Communities on Hydrocarbons by Means of Laser Scanning Microscopy.” In Hydrocarbon and Lipid Microbiology Protocols–Ultrastructure and Imaging, edited by T. J. McGenity , K. N. Timmis , and B. Nogales Fernandez , 29–47. Springer.

[jemt70064-bib-0038] Neu, T. R. , and J. R. Lawrence . 2016b. “Laser Microscopy for the Study of Biofilms: Issues and Options.” In Aquatic Biofilms: Ecology, Water Quality and Wastewater Treatment, edited by A. M. Romaní , H. Guasch , and D. Balaguer , 29–45. Caister Academic Press.

[jemt70064-bib-0039] Neu, T. R. , B. Manz , F. Volke , J. J. Dynes , A. P. Hitchcock , and J. R. Lawrence . 2010. “Advanced Imaging Techniques for Assessment of Structure, Composition and Function in Biofilm Systems.” FEMS Microbiology Ecology 72: 1–21. 20180852 10.1111/j.1574-6941.2010.00837.x

[jemt70064-bib-0040] Neu, T. R. , G. D. W. Swerhone , and J. R. Lawrence . 2001. “Assessment of Lectin‐Binding Analysis for in Situ Detection of Glycoconjugates in Biofilm Systems.” Microbiology 147: 299–313. 11158347 10.1099/00221287-147-2-299

[jemt70064-bib-0041] Palmer, R. J. , J. Haagensen , T. R. Neu , and C. Sternberg . 2006. “Confocal Microscopy of Biofilms–Spatiotemporal Approaches.” In Handbook of Biological Confocal Microscopy, edited by J. B. Pawley , 882–900. Springer.

[jemt70064-bib-0042] Pawley, J. B. 2006. Handbook of Biological Confocal Microscopy. Springer.

[jemt70064-bib-0043] Power, R. M. , and J. Huisken . 2017. “A Guide to Light‐Sheet Fluorescence Microscopy for Multiscale Imaging.” Nature Methods 14, no. 4: 360–373. 28362435 10.1038/nmeth.4224

[jemt70064-bib-0044] Russ, J. C. , and F. B. Neal . 2016. The Image Processing Handbook. CRC Press.

[jemt70064-bib-0045] Savidge, T. , and C. Pothoulakis . 2004. Microbial Imaging. Elsevier.

[jemt70064-bib-0046] Scheele, C. L. G. J. , D. Herrmann , E. Yamashita , et al. 2022. “Multiphoton Intravital Microscopy of Rodents.” Nature Reviews Methods Primers 2, no. 1: 89. 10.1038/s43586-022-00168-w PMC10449057 37621948

[jemt70064-bib-0047] Schermelleh, L. , R. Heintzmann , and H. Leonhardt . 2010. “A Guide to Super‐Resolution Fluorescence Microscopy.” Journal of Cell Biology 190, no. 2: 165–175. 20643879 10.1083/jcb.201002018 PMC2918923

[jemt70064-bib-0048] Sheppard, C. J. R. , and D. M. Shotton . 1997. Confocal Laser Scanning Microscopy. Bios Scientific Publishers.

[jemt70064-bib-0049] Siegumfeldt, H. , K. B. Rechinger , and M. Jakobsen . 1999. “Use of Fluorescence Ration Imaging for Intracellular pH Determination of Individual Bacterial Cells in Mixed Cultures.” Microbiology 145: 1703–1709. 10439409 10.1099/13500872-145-7-1703

[jemt70064-bib-0050] Sluder, G. , and D. E. Wolf . 2013. Digital Microscopy. Academic Press. 10.1016/B978-0-12-407761-4.09952-8 23931526

[jemt70064-bib-0051] Stelzer, E. H. K. , F. Strobl , B.‐J. Chang , et al. 2021. “Light Sheet Fluorescence Microscopy.” Nature Reviews Methods Primers 1, no. 1: 73.

[jemt70064-bib-0052] Torno, K. , B. K. Wright , M. R. Jones , M. A. Digman , E. Gratton , and M. Phillips . 2013. “Real‐Time Analysis of Metabolic Activity Within *Lactobacillus acidophilus* by Phasor Fluorescence Lifetime Imaging Microscopy of NADH.” Current Microbiology 66, no. 4: 365–367. 23233088 10.1007/s00284-012-0285-2

[jemt70064-bib-0053] van Aarle, I. M. , P. A. Olsson , and B. Söderström . 2001. “Microscopic Detection of Phosphatase Activity of Saprophytic and Arbuscular Mycorrhizal Fungi Using a Fluorogenic Substrate.” Mycologia 93: 17–24.

[jemt70064-bib-0054] Venugopalan, V. P. , M. Kuehn , M. Hausner , D. Springael , P. A. Wilderer , and S. Wuertz . 2005. “Architecture of a Nascent Sphingomonas sp. Biofilm Under Varied Hydrodynamic Conditions.” Applied and Environmental Microbiology 71: 2677–2686. 15870359 10.1128/AEM.71.5.2677-2686.2005 PMC1087527

[jemt70064-bib-0055] Volpi, E. V. , and J. M. Bridger . 2008. “FISH Glossary: An Overview of the Fluorescence in Situ Hybridization Technique.” BioTechniques 45, no. 4: 385–409. 18855767 10.2144/000112811

[jemt70064-bib-0056] Waharte, F. , K. Steenkeste , R. Briandet , and M. P. Fontaine‐Aupart . 2010. “Diffusion Measurements Inside Biofilms by Image‐Based Fluorescence Recovery After Photobleaching (FRAP) Analysis With a Commercial Confocal Laser Scanning Microscope.” Applied and Environmental Microbiology 76, no. 17: 5860–5869. 20639359 10.1128/AEM.00754-10 PMC2935062

[jemt70064-bib-0057] Walczysko, P. , U. Kuhlicke , S. Knappe , C. Cordes , and T. R. Neu . 2008. “In Situ Activity Measurement of Suspended and Immobilized Microbial Communities by Fluorescence Lifetime Imaging (FLIM).” Applied and Environmental Microbiology 74: 294–299. 17981940 10.1128/AEM.01806-07 PMC2223234

[jemt70064-bib-0058] Weber, M. , M. Leutenegger , S. Stoldt , et al. 2021. “MINSTED fluorescence localization and nanoscopy.” Nature Photonics 15, no. 5: 361–366. 33953795 10.1038/s41566-021-00774-2 PMC7610723

[jemt70064-bib-0059] Wilkinson, M. H. F. , and F. Shut . 1998. Digital Image Analysis of Microbes. Wiley.

[jemt70064-bib-0060] Yuste, R. 2011. Imaging: A Laboratory Manual. Cold Spring Harbour Laboratory Press.

[jemt70064-bib-0061] Zhang, J. , M. Zhang , Y. Wang , E. Donarski , and A. Gahlmann . 2021. “Optically Accessible Microfluidic Flow Channels for Noninvasive High‐Resolution Biofilm Imaging Using Lattice Light Sheet Microscopy.” Journal of Physical Chemistry B 125, no. 44: 12187–12196. 34714647 10.1021/acs.jpcb.1c07759 PMC8592114

[jemt70064-bib-0062] Zucker, R. M. 2006. “Evaluation of Confocal Microscopy System Performance.” Methods in Molecular Biology 319: 77–135. 16719352 10.1007/978-1-59259-993-6_5

[jemt70064-bib-0063] Zwirglmaier, K. 2005. “Fluorescence in Situ Hybridisation (FISH)–The Next Generation.” FEMS Microbiology Letters 246, no. 2: 151–158. 15899400 10.1016/j.femsle.2005.04.015

